# Multiple Different Defense Mechanisms Are Activated in the Young Transgenic Tobacco Plants Which Express the Full Length Genome of the Tobacco Mosaic Virus, and Are Resistant against this Virus

**DOI:** 10.1371/journal.pone.0107778

**Published:** 2014-09-22

**Authors:** Balaji Jada, Arto J. Soitamo, Shahid Aslam Siddiqui, Gayatri Murukesan, Eva-Mari Aro, Tapio Salakoski, Kirsi Lehto

**Affiliations:** 1 Department of Biochemistry, Laboratory of Molecular Plant Biology, University of Turku, Turku, Finland; 2 Department of Agricultural sciences, University of Helsinki, Helsinki, Finland; 3 Department of Information Technology, University of Turku, Turku, Finland; Zhejiang University, China

## Abstract

Previously described transgenic tobacco lines express the full length infectious Tobacco mosaic virus (TMV) genome under the 35S promoter (Siddiqui et al., 2007. Mol Plant Microbe Interact, 20: 1489–1494). Through their young stages these plants exhibit strong resistance against both the endogenously expressed and exogenously inoculated TMV, but at the age of about 7–8 weeks they break into TMV infection, with typical severe virus symptoms. Infections with some other viruses (Potato viruses Y, A, and X) induce the breaking of the TMV resistance and lead to synergistic proliferation of both viruses. To deduce the gene functions related to this early resistance, we have performed microarray analysis of the transgenic plants during the early resistant stage, and after the resistance break, and also of TMV-infected wild type tobacco plants. Comparison of these transcriptomes to those of corresponding wild type healthy plants indicated that 1362, 1150 and 550 transcripts were up-regulated in the transgenic plants before and after the resistance break, and in the TMV-infected wild type tobacco plants, respectively, and 1422, 1200 and 480 transcripts were down-regulated in these plants, respectively. These transcriptome alterations were distinctly different between the three types of plants, and it appears that several different mechanisms, such as the enhanced expression of the defense, hormone signaling and protein degradation pathways contributed to the TMV-resistance in the young transgenic plants. In addition to these alterations, we also observed a distinct and unique gene expression alteration in these plants, which was the strong suppression of the translational machinery. This may also contribute to the resistance by slowing down the synthesis of viral proteins. Viral replication potential may also be suppressed, to some extent, by the reduction of the translation initiation and elongation factors eIF-3 and eEF1A and B, which are required for the TMV replication complex.

## Introduction

Viruses are obligate intracellular molecular parasites which depend on host's cellular machinery and on multiple host factors to complete their infectious life cycle. They utilize a large variety of host-encoded proteins and molecular structures as components of their replication complex or cell-to-cell movement machinery [1]–[6] and various cellular compartments (typically various membranous structures) as their replication sites [1], [7]–[10]. For instance, many RNA-viruses use the host's translation elongation factor 1A (eEF1A) as a component of their replication complex, tobamoviruses use also the factors eEF1B and eIF3 in this complex, and potyviral VPg molecules interact with the host's initiation factor 4E (eIF4E) for promoting their translation [11]–[19].

Viruses can also alter the functions and composition of their host cells to benefit their own proliferation. For instance, they can enhance the expression of their needed host factors, bind or suppress various resistance factors, induce changes in the lipid composition of infected cells, and interfere with host's hormonal pathways [1], [8], [13], [20]–[22].

Viruses can initiate infection process only in susceptible host species that provide compatible host factors, needed for the viral replication and spread. However, many of these potential host species also recognize the invading viruses and mount different defense mechanisms to stop their proliferation or spread. For instance, activation of the R-gene mediated resistance leads to hypersensitive reaction (HR) and virus localization, enhanced expression of various defense-related genes and induction of systemic acquired resistance (SAR) [23]–[25]. Accumulation of virus-specific double-stranded RNAs also induces the RNA-silencing mediated immune-system in plants [26], which leads to sequence-specific degradation of the viral RNAs. To counteract these silencing-mediated defense reactions, viruses produce specific silencing suppressor proteins (Viral RNA-silencing suppressors, VRSS) which interfere with different steps of the silencing pathways [22], [27]–[31]. Some of these VRSS-factors have been identified as viral host determinants or pathogenicity factors within specific host species [32]–[35], demonstrating the importance of this defense/counter-defense interaction between viruses and their hosts. The VRSS factors may also interfere with the silencing-mediated endogenous regulatory pathways in the cells [36]–[39]. This may happen as a mere side-effect of the viral counter-defense, or as an active means to weaken hosts' cellular status.

The virus-host interactions thus consist of a very complex molecular interplay. It involves depletion of various host factors and energy compounds through viral parasitism, altered expression of the viral-induced host factors, active defense mechanisms mounted by the host, active viral counter-defense mechanisms, disturbance of the silencing-mediated regulatory network, and the general infection related stress-reaction in the host [17], [23], [40]. Some of these interactions depend on functions of individual virus-encoded genes, while others are related to the replication of the viral RNA or to the consorted action of various viral products. They may lead to either plant resistance, or to virus proliferation and symptom development in the host plant.

Now, different system biology approaches are available to investigate the plant responses induced in different stages of virus infections, or in transgenic plants that express individual viral genes. Expression levels of many hundreds or even several thousands of genes have been found to be altered in these plants, and although these alterations have some common features (enhanced expression of defense- and stress-related genes), they are mostly unique and specific to each virus/host combination [20], [21], [41]–[47]. This illustrates that the molecular interactions are unique and specific in each compatible or incompatible virus-host combination.

Through the last few decades, Tobacco mosaic virus (TMV) or its constituent genes have often been used as a model system to investigate the plant-virus interactions and to dissect the details of viral replication, movement, host resistance and physiological alterations [6], [19], [42], [48]–[52]. Here we are using the functional genomics to study the molecular response of transgenic tobacco plants expressing the infectious TMV genome under the constitutive 35S promoter [53]. Interestingly, during the early growth stages (up to about 7–8 weeks after germination) these plants accumulate only a very low level of the TMV RNA, and also exhibit strong resistance against external TMV infections. After this period the resistance breaks, plants become infected from the transformed TMV sequence, accumulate high levels of TMV RNA and show typical TMV symptoms. To identify the gene functions that are associated to this early resistance we have conducted a microarray analysis of these transgenic plants just before resistance break (BRB), and compared their gene expression patterns to those of corresponding healthy wild type (wt) plants. The observed trasncriptome alterations were compared to those observed in the same plants after resistance break (ARB) and in TMV-infected (TMVi) wt tobacco plants. Gene expression alterations were also compared to those observed in other transgenic tobacco plants expressing various virus-derived VRSS genes [54] that are known not to be resistant against TMV, to reveal the genes or processes that would be specific to the resistance condition.

## Results

**Figure 1:**
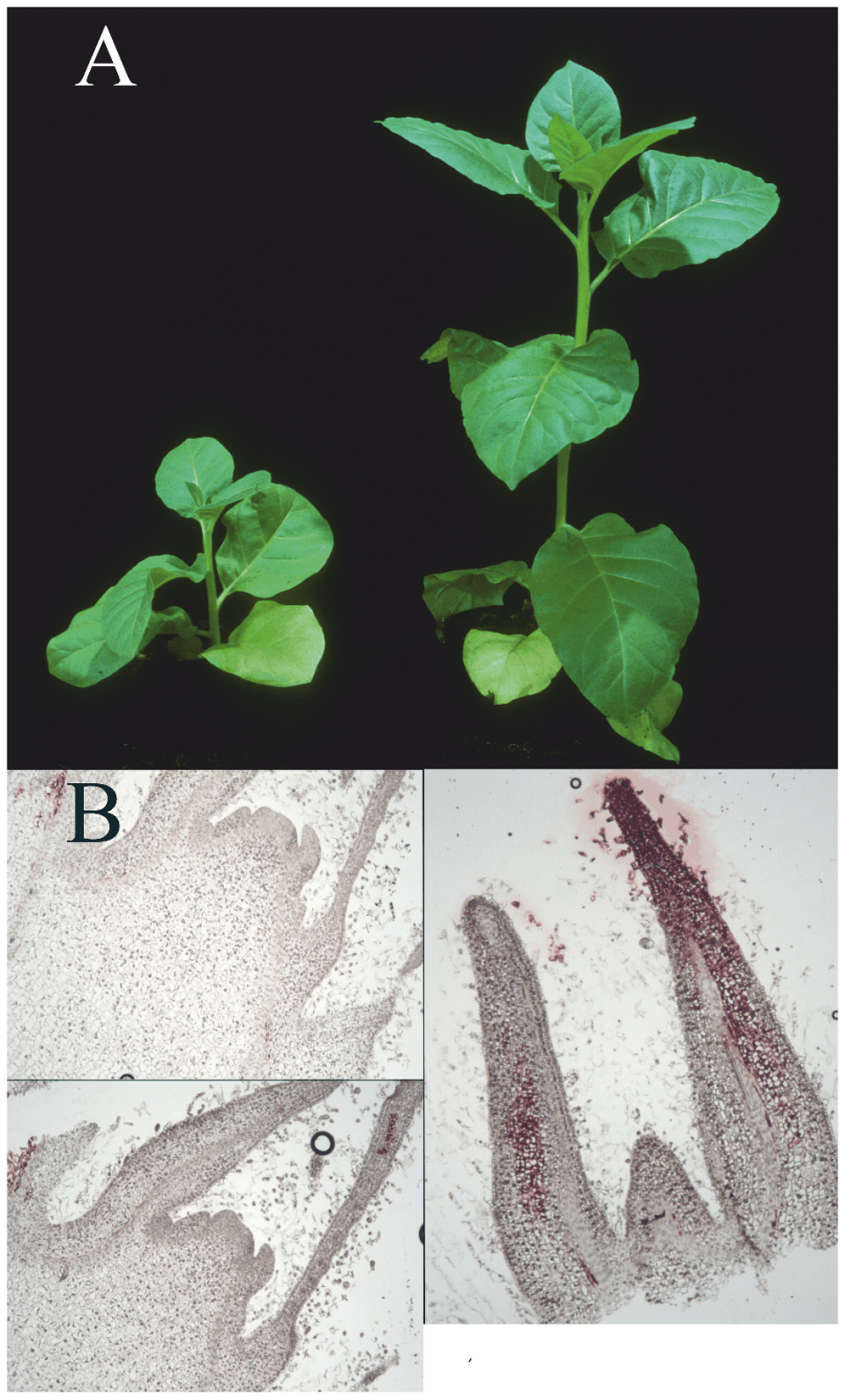
Phenotype of the transgenic plants that harbor the full length infectious TMV cDNA, under 35S promoter. (A) BRB-TMV transgenic plant (on the left), at the age of six weeks after germination. The plants show no viral symptoms, and do not contain any detectable viral RNA or CP, but they are severely stunted as compared to the wild type plants of the same age. (B), Anti-TMV CP labeled thin sections of the apical domain of the TMV transgenic plant during the early stage of the resistance break (at 8 weeks after germination). Isolated TMV-positive foci are detected in the vascular tissue, and in the tips of leaf initials. On the left, two adjacent sections are shown to illustrate the small size of the foci.

Transgenic tobacco plants that express the wt TMV genome have been previously produced and characterized in our laboratory [53]. All transgenic lines derived from separate transformation events, and sibling lines from the same transformations all had the same, consistent, stunted phenotype (Figure 1A), were initially resistant against TMV, and broke into a strong TMV infection typically at about 7–9 weeks after germination. This emerging infection verified the positive transcription of the transgene. Positive transgene expression status, even before the resistance break, was also shown by positive, although very low detection of the viral RNA by northern blotting and by RT-PCR. At the resistance breaking stage, the TMV-coat protein positive cells, as detected by *in situ* immunolabeling, first appeared as isolated infection foci in the vascular tissues of the upper leaves (Figure 1B). Typical viral symptoms also first appeared on upper leaves of the plants and then slowly progressed towards the lower older leaves, i.e. showing similar symptom pattern as a normal TMV-infection in wt tobacco plants (data not shown).

To investigate the molecular mechanisms underlying this resistant condition, transcriptomic profiles of three young resistant plants (BRB), and of the same plants after the resistance break (ARB), and of three wt TMV-infected (TMVi) plants were analyzed by the microarray approach, and compared each to the transcriptomes of healthy control plants of corresponding age. The observed transcriptome alterations were compared between these three types of plants to reveal gene functions that would be specific to each condition.

The microarray analysis was performed using Tobacco 4×44K microarray (Agilent), according to Agilent's standard procedures (see Methods). Our previous microarray analyses of different transgenic tobacco lines have revealed that the transcript profiles of control plants transformed with the empty pBin61 transformation vector are approximately equal to those of the wild type healthy plants [41], and therefore only the wild type plants were used here as controls. The raw microarray data was normalized and subjected to statistical analysis, with BH false discovery rate of less than 0.05 (Student t-test with adjusted P-value <0.05). Subsequently, the transcripts that were 2-fold up- or down-regulated, as compared to the corresponding healthy control plants, were considered as differentially expressed in the test plants. Some of the expression levels of randomly selected genes were verified by using RT-qPCR, with essentially same results as was attained with the microarray method (Table 1).

**Table 1:** Microarray results verification by using quantitative real-time PCR (RT-qPCR).

		Log value	Log value	s.e. of C_t_
EST/mRNA	Description	Microarray	RT-qPCR	of RT-qPCR
**BRB transgenic plants**	**up-regulated transcripts**			
EB683763	P-rich protein NtEIG-C29	2.9	3.0	0.46
BP128776	DNAJ heat shock protein	4.6	6.8	0.21
**BRB transgenic plants**	**down-regulated transcripts**			
CV018266	60 s acidic ribosomal protein-like protein	−2.0	−2.4	0.15
DV158570	40S ribosomal protein S8	−2.1	−2.6	0.11
EB683199	60S ribosomal protein L35	−2.1	−2.8	0.03
**ARB transgenic plants**	**up-regulated transcripts**			
EB438730	Dicer-like 2 protein (DCL901)	3.1	3.3	0.53
EH620111	Pathogenesis-related protein 1B precursor	2.6	3.8	0.24
EH617029	WRKY transcription factor-30	1.0	1.1	0.03
**ARB transgenic plants**	**down-regulated transcripts**			
CV017513	Chlorophyll a-b binding protein 3A	−5.1	−5.3	0.47
EH620344	F box related protein	−1.2	−1.1	0.09
**TMV infected plants**	**up-regulated transcripts**			
EH620111	Pathogenesis-related protein 1B precursor	3.5	3.6	0.15
EB643469	60 s Acidic ribosomal protein	4.5	6.0	0.57
**TMV infected plants**	**down-regulated transcripts**			
EH620344	F box related protein	−1.5	−1.7	0.22
CV017513	Chlorophyll a–b binding protein 3A	−1.8	−1.9	0.22
TA12913_4097	Pollen coat like protein	−3.5	−4.7	0.29

Accumulation of some up- or down-regulated transcripts of the the wild type, BRB- and ARB- transgenic, and of TMV infected wt plants were tested by RT-qPCR, and compared to their accumulation levels observed by the microarray assay. The depicted log values are normalized mean intensive value (n = 3) differences of the wt control plants and the different plant types of TMV-transgenic and TMV-infected wt plants. Statistical significance of the results was tested using Student's t-test (p<0.05). The Standard error of mean (s.e) is calculated for the C_t_ values of the RT-qPCR results.

**Figure 2:**
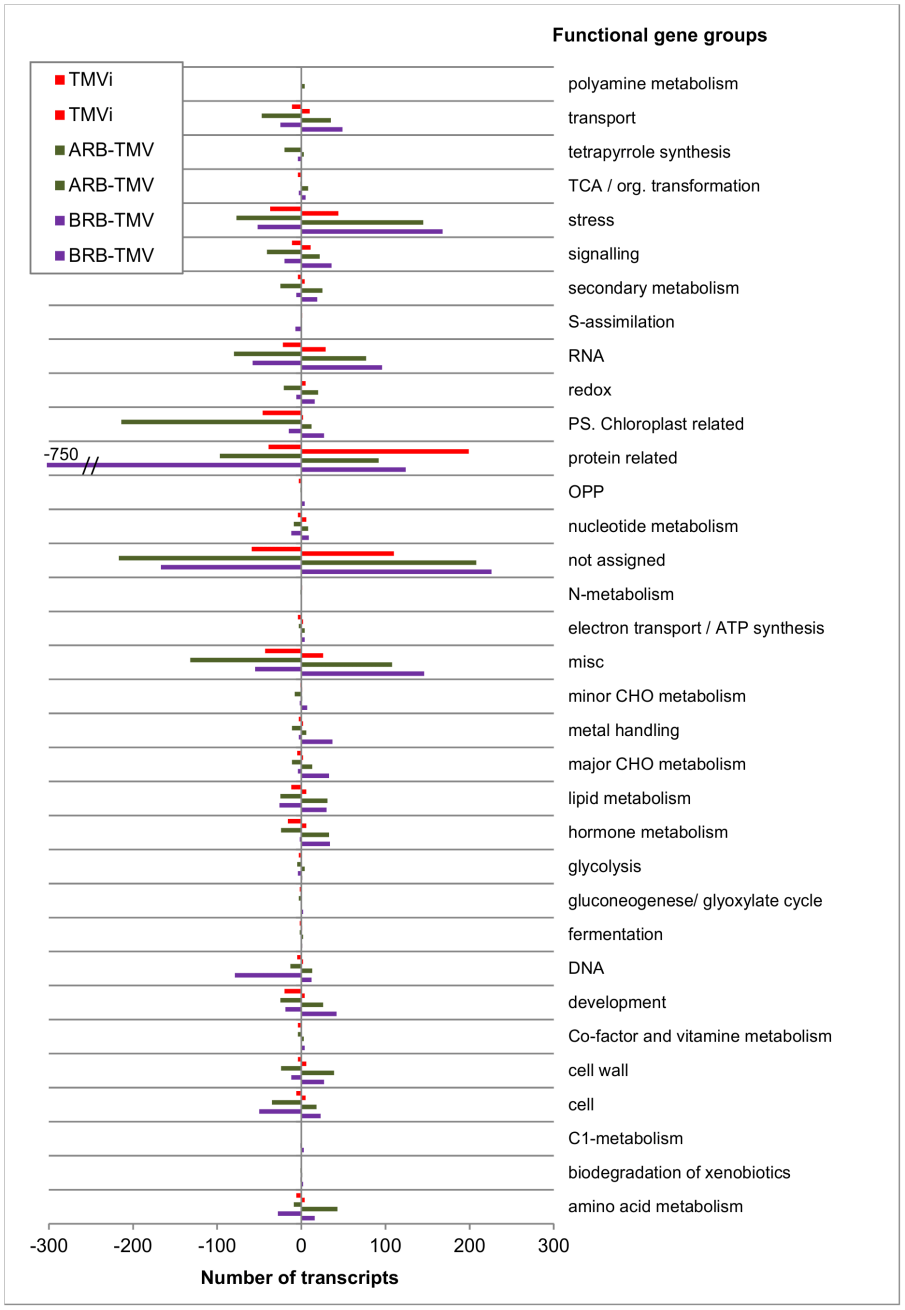
A overview chart showing the distribution in different functional groups of the up- and down-regulated transcripts and their numbers in the BRB- and ARB-TMV transgenic, and in TMVi tobacco plants.

The microarray analyses indicate that total of 1362 transcripts were up- and 1422 transcripts were down-regulated in the BRB transgenic plants (Figure 2, Table S1), total of 1150 transcripts were up- and 1200 were down-regulated in the ARB transgenic plants (Figure 2, Table S2), and 550 transcripts were up- and 480 transcripts down-regulated in the TMVi plants, respectively (Figure 2, Table S3). The transcriptional alterations were very distinct and different between these three types of plants. The details of these alterations are compared and discussed in further sections.

### Transcripts of the protein synthesis machinery were strongly reduced in BRB plants

Many viruses infecting eukaryotic hosts use different mechanisms to reduce the host-specific protein synthesis, and to increase the synthesis of the virus-specific proteins [55]. Interestingly, the three different types of plants examined in this study (BRB and ARB transgenic plants and TMVi tobacco plants) showed strong – but strikingly different - alterations in the expression levels of the translation-related transcripts.

In BRB transgenic plants, the largest group of down-regulated genes (total of 750) was composed of translation-related transcripts (Figures 2, S1, Table S4). Most of them were pertained to the cytosolic ribosomal genes, including transcripts coding for various 60S ribosomal proteins (total of 391 transcripts, e.g. the L5–L15, L17–L39, L44, P0, P1, P3 and P4), and for various 40S ribosomal proteins (total of 222 transcripts, e.g. S3–S29), with reduction levels ranging up to 5,5-fold level. Also, some transcripts related to the 18S ribosomal RNA and ribosomal biogenesis regulators were down-regulated, as were several (total of 33) transcripts coding for translation initiation factors (including eIF2γ, eIF-4A, eIF-3 genes), and for elongation-related genes like nascent polypeptide complex, LOS1 and elongation factors eEF1A, B, P, Δ and TuA. Also, several transcripts related to the post-translational modifications, protein targeting (such as TIC40- and TOC75-complexes), amino acid biosynthesis and various protein folding chaperons were down-regulated (Table S4) in the BRB-plants. In addition, about 150 protein processing and degradation-related transcripts were clearly up-regulated in the BRB-plants, including ubiquitin ligases and conjugating proteins, autophagy 8c proteins and a variety of peptidases and proteases. Also, some transcripts coding for translation initiation factors, amino acid synthesis, protease inhibitors, ribosomal proteins and protein kinases were up-regulated (Table S5).

Interestingly, nearly opposite expression pattern of translation-related genes was observed in the ARB transgenic plants. In these plants, only 120 translation-related transcripts were down-regulated, and a total of 135 translation-related transcripts were up-regulated (Table S5). Among these, several transcripts related to the ribosomes and translational initiation (total of 13), amino acid biosynthesis (total of 25), and protein secreting pathways (6) were induced. In contrast to the BRB-plants, 60 transcripts coding for proteases, peptidases and ubiquitin-mediated protein degradation pathways were down-regulated (Table S4), including the transcripts for the S41 and M48 peptidases, believed to be involved in the processing of the D1 protein of the photosystem II (PSII) in plants [56]–[58]. Also, several genes related to transcription (e.g. sigma factors and RNA polymerases), or to the posttranslational modifications were down-regulated. Only 16 genes coding for different proteases and 29 genes coding for ubiquitin-mediated protein degradation pathway were up-regulated. Some of the up-regulated transcripts in the ARB transgenic plants were related to metallocarboxypeptidase, kinases and amino acid degradation.

In the TMVi plants, the translation and protein processing related transcripts were predominantly up-regulated, with a total of 197 translation-related transcripts being up-regulated and 44 being down-regulated (Table S4 & S5). Most interestingly, many of the up-regulated transcripts in the TMVi plants were related to 60S (114) and 40S (55) ribosomes and their subunits, and to translation elongation, which were down-regulated in the BRB transgenic plants; these transcripts were enhanced by 2–23.2 fold. Also, some transcripts related to, nucleus targeting, protein folding and protein phosphatases, and protein and amino acid degradation pathways were up-regulated in the TMVi plants, including ubiquitin mediated pathway and several proteases and peptidases. Only 7 transcripts related to ribosomal proteins and 26S ribosomal RNA, and 11 transcripts related to post-translational modification pathways were down-regulated in TMVi plants.

### Many stress-related genes were induced in TMV-transgenic plants but not in wild type TMV infected tobacco plants

In compatible plant-virus interactions the virus must be able to infect the host plant without mounting excessive or fast defense reactions, or to suppress the basal host defense mechanisms [50], [59], [60]. The interaction of TMV with tobacco is known to be compatible in nature [50]. In this work such full compatibility was found only in TMVi wild type tobacco plants where only few biotic and abiotic stress response-related transcripts were induced. Instead, the TMV- expressing BRB and ARB transgenic plants showed strongly activated defense-responses.

In the BRB transgenic plants, total of 174 stress response-related transcripts were up-regulated and 76 were down-regulated (Figure S2, Table S7 & S6). Many of the up-regulated transcripts were coding for heat shock proteins (20), chitinases (17), elicitor inducible proteins (16), HR-proteins (12), glycine rich proteins (13), osmotin precursors (8), wound responsive proteins (16) and disease resistance proteins (6). Interestingly, also the non-functional allele of the TMV-resistance gene N, 13 SAR- and 16 HR-related transcripts were also enhanced in the BRB plants [59], [61], even that no visible signs of HR was seen on the plants. Several transcripts related to various abiotic stresses such as cold acclimation, dehydration, reactive oxygen species such as peroxidases, and to various redox-reactions, as well as transcripts coding for cytochrome p450 and glutathione transferases were also up-regulated in these plants (Table S7). On the other hand, several other transcripts pertaining to heat shock proteins, defense proteins, ATP binding proteins, arabinogalactan proteins, peroxidases and cytochrome p450 were down-regulated in BRB plants (Table S6).

Even more (total of 192) of stress-related genes were up-regulated in the ARB transgenic plants (Table S7), including transcripts coding for heat shock proteins (25), PR-proteins (17), chitinases (12), dehydration response factors (8), different protease inhibitors (10), peroxidases (18), redoxins (11) and various defense proteins (23) such as defensins, thaumatin, thionins and germins. Also, the transcripts of the TMV viral coat protein transcript, which was completely absent in BRB transgenic plants, accumulated to very high levels in the ARB transgenic plants. Various stress-related transcripts (total of 131), e.g. coding for heat shock proteins, wound induced proteins, elicitor inducible proteins, methanol inducible proteins, NDR1-like, cytochrome p450, thioredoxins and glutathione transferases were down-regulated in the ARB transgenic plants (Table S6). Interestingly, SAR-proteins (6) and HR- (3) related transcripts were down-regulated in the ARB transgenic plants in contrast to their up-regulation in the BRB transgenic plants.

In TMVi plants, only total of 61 of stress-related transcripts were up-regulated (Table S7). These included several transcripts coding for heat shock proteins (12), chitinases (9), pathogenesis related proteins (11) and senescence associated proteins (2). The transcript for the viral coat protein was highly expressed in the TMV infected plants. A total of 40 stress-related transcripts, e.g. coding for heat shock proteins, wound induced proteins, glutathione-S-transferase and peroxidases were down-regulated in the TMVi plants as well (Table S6).

### Hormone and development related transcripts

Plant hormones play a major role in various defense signaling pathways, and some viruses interact with these signaling pathways to enhance their infection process [14], [17], [18]. Our microarray data also indicates differential hormonal regulation between the BRB and ARB transgenic plants, and the TMVi plants. In the BRB transgenic plants, total of 60 transcripts related to hormones and development were up-regulated. Particularly the auxin repressed/dormancy associated, and auxin- and ethylene responsive transcripts were up-regulated by 10–15 fold (Tables S8 & S9). Also several other hormones and development-related transcripts were either down- or up-regulated (Tables S8 & S9) in these plants, including transcripts related to ethylene, senescence, and abscisic acid synthesis or to various development associated genes.

In ARB plants, a total of 59 transcripts related to defence hormones (ethylene and jasmonic acid) or coding for development-related embryo-specific proteins were up-regulated (Table S9). The jasmonic acid-related genes were induced by up to 26-fold in these plants. Interestingly, 8 transcripts related to auxin repressed/dormancy, auxin associated and SAUR-families were down-regulated, in contrast to their up-regulation in the BRB transgenic plants. Some transcripts related to ethylene synthesis, abscisic acid, gibberellin 20-oxidase, LEA proteins and pentatricopeptide repeat-containing proteins were down- regulated in these plants (Table S8).

In TMVi plants, a total of 33 transcripts related to hormones and development were down-regulated (Table S8), including some transcripts coding for auxin repressed genes, ethylene signal transduction, gibberellin oxidase, jasmonic acid and senescence related genes. A few transcripts related to hormones and development (total of 5) including transcripts coding for ethylene biosynthesis, protodermal factor and pale cress related were up-regulated (Table S9).

### Photosynthesis and carbohydrate metabolism related transcripts

Several studies indicate that virus infections or expression of virus-derived genes in transgenic plants reduce the photosynthesis process and cause alterations of the carbohydrate metabolism and translocation in the plants [17], [41], [43], [44], [62], [63]. Furthermore, some chloroplast proteins (i.e. Rubisco activase, ATP-synthase γ-subunit, and the 33K subunit of the oxygen evolving complex) interact directly with the TMV-encoded replicase protein. These proteins mediate some level of suppression of virus replication, while the virus infection causes some suppression of their expression [51], [64]. Interestingly, in the three types of our studied plants, fewer photosynthesis and carbohydrate metabolism-related transcripts were down-regulated in the BRB plants as compared to the ARB and TMVi plants. Only total of 13 transcripts coding for chloroplast proteins, Rubisco activase, NADP-dependent g-3-p dehydrogenase, plastocyanin, ferredoxin and tetrapyrrole synthesis were down-regulated in the BRB plants (Table S10). Similarly, only few photosynthesis-related transcripts (total 28), coding for PSI and II subunits L, O and R, OEC proteins, PGR5-1A and alternative oxidase, or related to chlororespiration and NADPH dehydrogenase complex were up-regulated in these plants (Table S11).

Contrastingly, total of 239 photosynthesis-related genes were down-regulated in the ARB transgenic plants (Table S10). Many of these (total of 227) were related to the photosynthetic machinery, as they coded for the chlorophyll binding and synthesis related proteins (125), or for the subunits of PSI and II, and of the OEC (including the 33 kDa subunit), and for plastocyanin and PGR5-1A. A total of 48 transcripts related to carbon metabolism (i.e. related to starch degradation, sucrose synthesis, Calvin cycle or photorespiration, or coding for electron carriers, carbonic anhydrase or enolase) were up-regulated in ARB plants (Table S11).

Similar to the ARB transgenic plants, photosynthesis related transcripts were predominantly down-regulated in TMVi plants (Table S10). Total of 55 down-regulated transcripts were related to photosynthesis and carbohydrate metabolism (i.e. coding for the chlorophyll a, b binding proteins, components of the PSI and PSII complexes, PGR5-like, carbonic anhydrase, glycolysis, Ribulose biphosphate carboxylase, and for the OEC components, including the 33 kDa protein). A few transcripts (total of 5) related to carbohydrate metabolism (including β-amylase, trehalose-6-phospahte phosphatase and L-lactate dehydrogenase encoding transcripts) were up-regulated in the TMV-infected plants (Table S11).

### Cell division, cell organization and DNA binding and repair related transcripts

The gene expression related to cell cycle and cell organization was differentially altered in BRB and ARB transgenic plants and in TMVi plants. Specific to the BRB transgenic plants, 30 and 18 transcripts related to cell cycle and organization were up- and down- regulated, respectively (Table S13 & S12). Specifically, several transcripts of B-type cyclins, peptidyl-prolyl cis-trans isomerases, mitotic spindle check proteins, XKLP2 targeting protein, Knolle protein, and various nucleolus and transport-related proteins were down-regulated, while some transcripts related to cell division and cell organization, including annexins, HIPL2, myosin-13 and tubulins were up-regulated in these plants.

In the ARB transgenic plants total of 36 transcripts related to cell cycle and cell organization were down-regulated (Table S12), including some transcripts coding for cell division control protein 48, tubulins, ankyrin repeat proteins, kinesin and vesicle transport realated proteins. Meanwhile, a total of 17 transcripts coding e.g. for cyclins, cell cycle check point control proteins, motor proteins and cell division inhibited proteins were up-regulated in these plants (Table S13). The cell division check point control protein RAD9A, which is abnormally expressed in several cancer types in animal cells [65], [66] was induced by 138× fold, as compared to the wild type controls.

Very few of cell division and organization-related transcripts were altered in the TMVi plants. Only 5 transcripts, coding for the CDC kinase and annexin were down-regulated (Table S12), and 5 transcripts related to cell cycle check point control protein and other miscellaneous cell cycle proteins were up-regulated in these plants (Table S13). Interestingly, the transcript of the cell division check point control protein RAD9A accumulated to 138× fold level also in the TMVi plants, as it did in the ARB transgenic plants.

Similar to the cell cycle and organization gene expression pattern, also the transcripts for histones and DNA repair proteins were affected differently in BRB and ARB transgenic plants and in TMVi plants. In the BRB transgenic plants, a total of 54 histone-encoding transcripts and 28 DNA binding and repair protein-encoding transcripts were down-regulated (Table S12), while 11 transcripts related to DNA repair and binding were up-regulated in these plants (Table S13). In the ARB transgenic plants, only 13 DNA binding protein transcripts were down-regulated, and 16 transcripts coding for various DNA binding proteins were up-regulated (Tables S12 & S13).

In TMVi plants, only four transcripts coding for DNA modifying proteins, i.e. one coding for a transposase, one coding for nuclease and two coding for DNA binding proteins were down-regulated, and only one NAP1-related transcript was up-regulated (Tables S12 & S13).

### Gene expression alterations unique for the BRB-TMV plants

**Figure 3:**
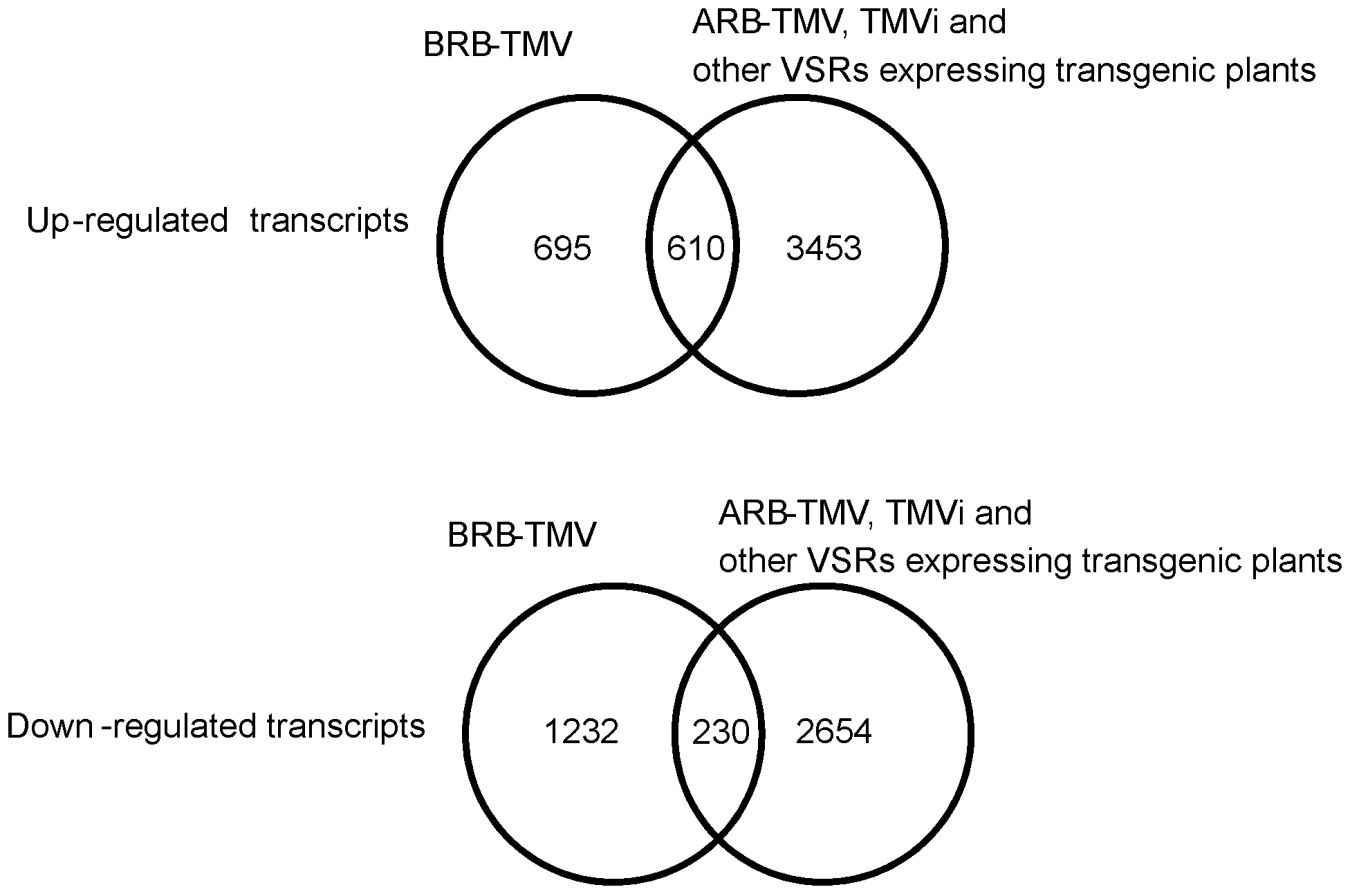
A Venn-diagram showing the numbers of up- and down-regulated transcripts that are either uniquely altered in the BRB-TMV transgenic plants, or in in the ARB-TMV transgenic plants or in the TMVi plants, or in the transgenic tobacco lines that express various viral silencing suppressors (as described in the text), and numbers of genes that are detected both in the BRB-TMV plants and in some of the other plants.

To reveal the gene expression alterations that were unique to the resistant stage of the BRB transgenic plants, their RNA expression profile was compared with that of the same transgenic plants at the ARB stage, with TMVi plants and with our previously published transcriptomes of transgenic plants expressing various viral silencing suppressors, i.e. HC-Pro from *Potato virus Y* [41], AC2 from *African cassava mosaic* virus [44], and P25 from *Potato virus* × [43], which all are known not to be resistant against TMV (data not shown). In total, 1305 up-regulated transcripts of the BRB transgenic plants were compared against 3453 up-regulated transcripts from the other transgenic and TMVi plants. This comparison revealed 695 unique and 610 commonly up-regulated transcripts in the TMV-resistant BRB transgenic plants (Figure 3). Many of the uniquely up-regulated transcripts in the BRB transgenic plants were related to stress (98), translation (88), photosynthesis (53), molecular transporters (27), lipids (27), hormones and development (40) and transcription factors (46) (Table S14).

Similarly, comparison of the total of 1462 transcripts that were down-regulated in the BRB transgenic plants against the 2884 down-regulated transcripts of other plants revealed that 1232 of these were unique to the BRB plants (Figure 3). Most of these unique down-regulated transcripts were related to translation (676), but some were related to chromatin (63), cell division (44), stress (48), and RNA processing (31) or were unknown for their function (159) in the BRB transgenic plants (Table S15). The strong suppression of protein synthesis machinery (see Figure S1) is a very special response observed only in this plant material, suggesting that it may be related to the unique virus resistance occurring in these plants. Still, the large number of transcripts that were distinctly altered (either up or down) in the resistance stage of the BRB plants suggests that many other functions may also contribut to the resistance.

### Not only transcripts but also total protein levels and profiles were different between BRB, ARB and TMVi plants

**Figure 4:**
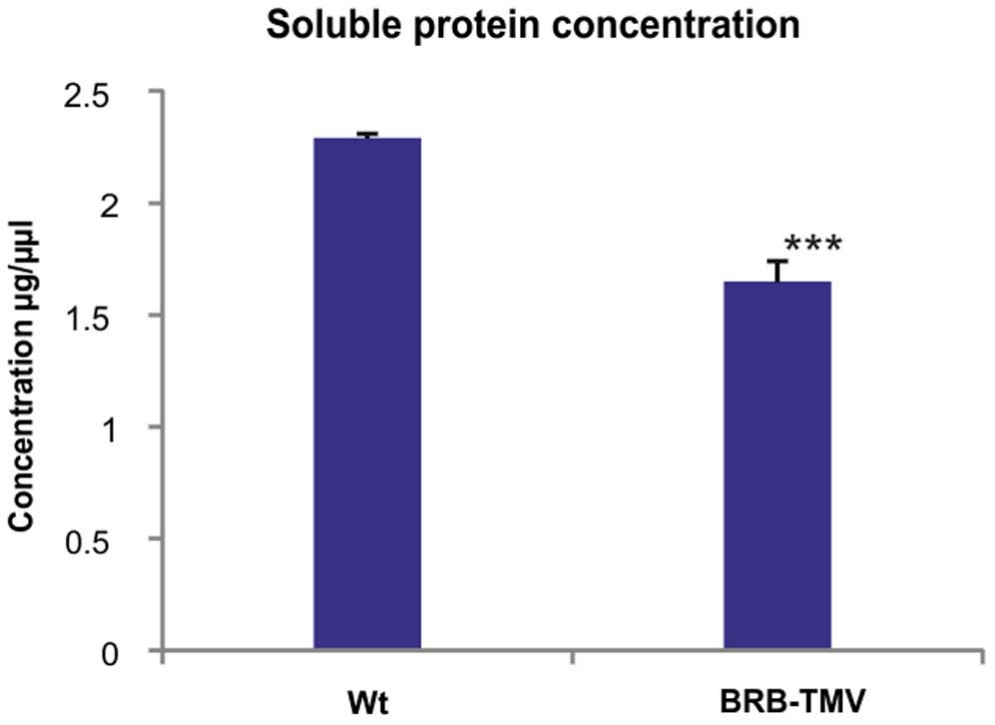
Lowry-quantitation of the soluble protein content of the BRB-TMV plants and of the corresponding wt control plants. Standard error of mean is presented as bars above the columns (consisting of three biological replicates). The confidence level determined by the Student's T-test, with confidence level higher than 95% is indicated by *, higher than 99% indicated by **, and higher than 0,001 indicated by ***.

To find out how the strong reduction of the proteins synthesis-related transcripts, and of the increase of protein degradation-related transcripts in the BRB-plants affects the total protein content of these plants, their total soluble protein was extracted, quantitated by the Lowry method, and compared to the soluble protein content of healthy control plants of the same age. This revealed that the total soluble protein content of the BRB-TMV transgenic plants was 28% lower that that of the wt plants (Figure 4). From previous literature it is known that the wt TMV infection does not significantly change the total protein content of tobacco leaves, although it significantly changes their protein composition [42], [51], [62], [64], [67]. We are not aware of any other virus infection condition where the total protein content would be reduced.

The total protein composition of the three types (BRB, ARB and TMVi) of test plants was analysed by using the 1D and 2D-PAGE, and compared with their corresponding wild type controls. The analyses were repeated with three biological replicates for each plant type. Upon loading of equal amounts of protein samples, the 2D-PAGE analysis showed that the protein profile of the BRB samples contained multiple altered (either enhanced or reduced) bands or spots as compared to the wild type control plants, but the altered spots were not identified (Figure S3). Also the samples of the ARB transgenic plants and TMV infected plants revealed reduction of multiple spots in the protein profiles compared to the healthy wild type plants, with strong accumulation of the TMV coat protein either as monomer (17,5 kDa) or as a dimer protein (35 kDa) Figure S3).

### TMV infection reduces photosynthetic oxygen evolution

**Figure 5:**
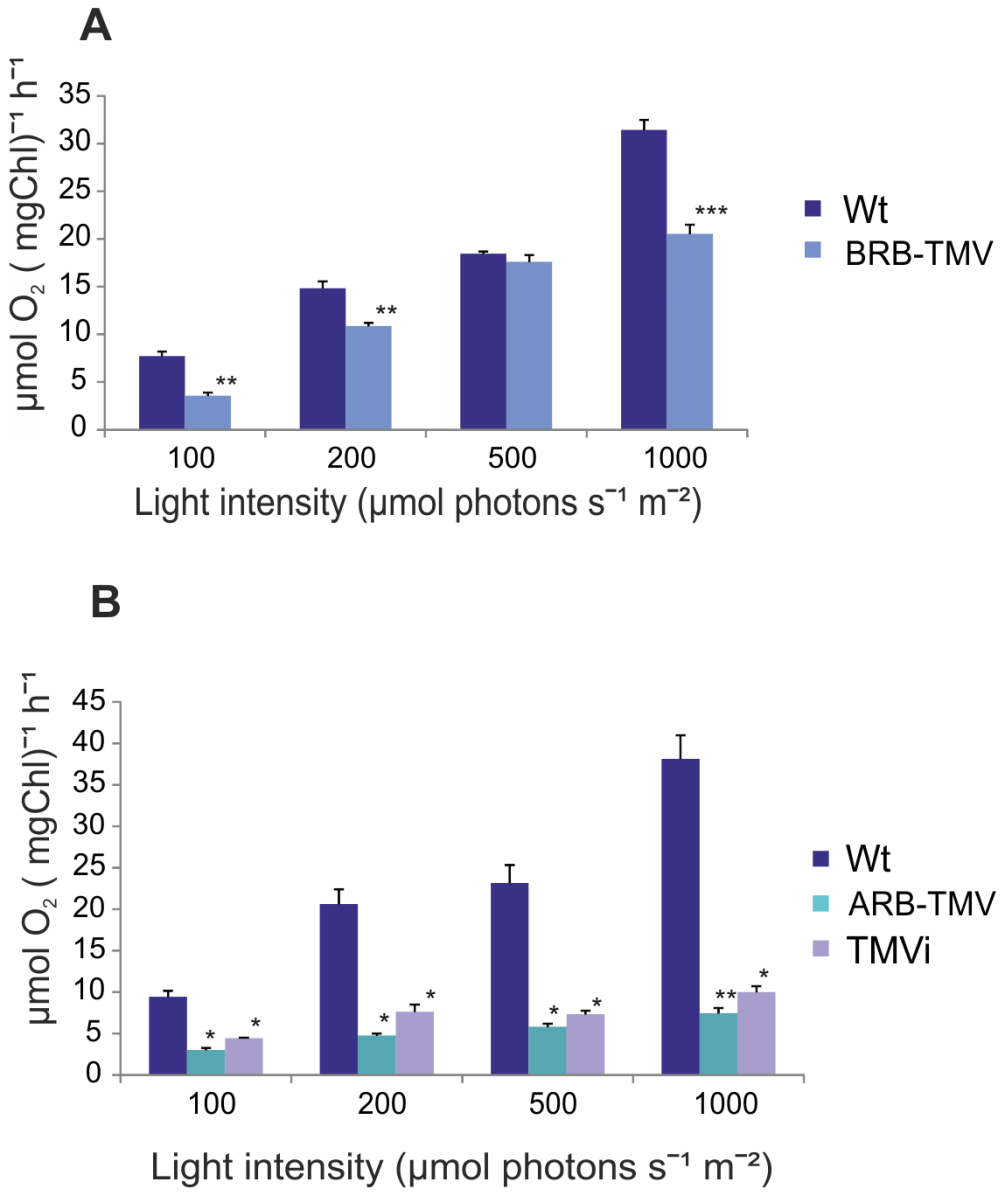
Light-responsive O_2_-evolution of photosystem II of the different test plants, as compared to the wild type control plants. BRB- (A), and ARB-TMV transgenic and TMVi plants (B). O_2_-evolution was measured of freshly isolated thylakoid membranes using DCBQ as an electron acceptor. Four light intensities is shown in x-axis (µmol photons s^−1^ m^−2^) and the O_2_-evolution in y-axis (µmol O_2_ (mgChl)^−1^ h^−1^). Standard error of mean is presented as bars above the columns (n = 4, consisting of five biological replicates). The confidence level determined by the Student's T-test, with confidence level higher than 95% is indicated by *, higher than 99% indicated by **, and higher than 99,9% indicated by ***.

Differential photosynthetic gene expression and appearance of the chlorotic TMV symptoms at different stages of plant growth indicated that photosynthetic activity was differentially altered in BRB and ARB transgenic plants and in TMVi plants. To analyze this, we measured their oxygen evolution per ug chlorophyll, and compared this against the oxygen evolution rate of control plants of the same age. The results indicated that oxygen evolution was not changed much in the BRB transgenic plants (Figure 5 A), whereas in ARB and TMVi plants it was strongly reduced (Figure 5 B). Thus, reduction of the photosynthetic activity was correlated to TMV virus accumulation.

### Response of the TMV-resistant plants to other viruses

**Figure 6:**
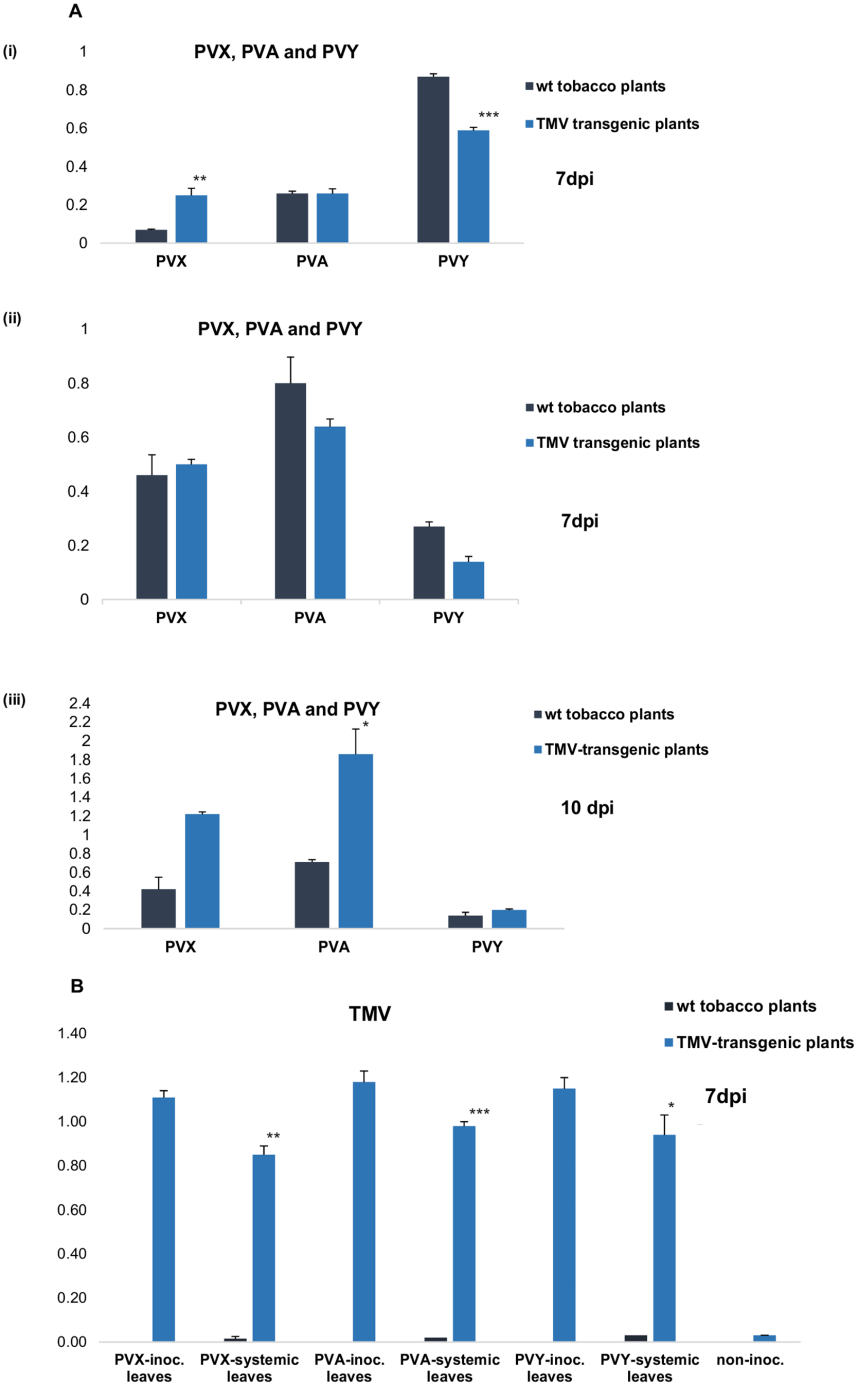
ELISA-mediated detection of the exogenously inoculated PVX, PVY and PVA viruses, and of the endogenously infecting TMV, from the BRB-TMV transgenic plants. A. Detection of PVX, PVY and PVA the from the inoculated leaves (i), and form the systemic leaves (ii) at 7 dpi (ii), and from the systemic leaves at 10 dpi (iii), and (B) detection of the TMV from the wt tobacco plants, and from the TMV-transgenic plants at 7 days after inoculation either with PVX, PVA and PVY. Standard error of mean is presented as bars above the columns (consisting of three biological replicates). The confidence level determined by the Student's T-test, with confidence level higher than 95% is indicated by *, higher than 99% indicated by **, and higher than 99,9% indicated by ***.

To check whether the observed TMV resistance in the young TMV-transgenic plants was TMV-specific, or active against a broader range of viruses, the plants were inoculated with PVX, PVY and PVA. ELISA results indicated that these viruses reacted somewhat differently to theTMV- transgenic host. The PVY level was somewhat increased, and PVX level somewhat decreased in the inoculated leaves of the transgenic plants, as compared to the inoculated leaves of the wt plants at 7 days post inoculation (dpi). Any of these viral levels did not differ significantly in the systemically infected leaves of TMV-transgenic and wt plants at 7 dpi, but PVA infection was strongly enhanced in the transgenic plants at 10 dpi (Figure 6). Meanwhile, these second infections also caused the breaking of the TMV-resistance and induced strong accumulation of the TMV in the transgenic plants, prior to the time of typical resistance break (Figure 6 B).

## Discussion

Typically, viruses can infect and complete their life cycle in a susceptible host and also easily spread systemically in such a host plant. This kind of compatible interaction involves effective use of necessary host factors and suppression of the host defense mechanisms [20], [50], [59]. In natural infections TMV has a very compatible interaction with its tobacco host. In inoculated mature tobacco leaves it typically spreads and accumulates evenly in all cells, while in systemically infected young leaves it spreads unevenly, causing mosaic where some tissues become fully infected, while others remain virus-free. These dark-green islands (DGI) are protected against the spreading infection by RNA-silencing, which becomes activated at the marginal regions of the initial infection foci [52].

Our transgenic tobacco plants represent an artificial inoculation system, where the infectious TMV genome is expressed under the constitutive 35S promoter, supposedly already in all cells of the germinating embryo. This leads to a very strong resistance condition that prevails in the plants until they reach a certain stage of maturity (about seven weeks from germination). This resistance was mostly specific to its endogenous inducer virus, although it provided low level transient protection also against two exogenously inoculated viruses (PVY and PVA), maybe due to the multiple defence genes that are activated in these tissues (Figure 6). However, as these viruses infected the plants, also the TMV-resistance was broken, indicating that the resistance mechanism was not durable under the stress factors, or was compromised by the silencing suppressors produced by the second viruses.

This suggests that the resistance may be related to the TMV-specific RNA silencing, and similar in nature to the resistance surrounding the DGIs in the young systemically infected leaves. Still, this was not supported by our earlier results, which showed that the TMV-derived transgene was not silenced by methylation, and that virus-specific siRNAs were not detected in the resistant tissues. The methylation level increased and the siRNAs became detectable only after the resistance break, indicating that the RNA silencing became activated at this stage [53].

The resistance was very strong in the young transgenic plants, and the viral RNA level remained very low: it was not detectable by Northern blotting [53] or by the microarray hybridization, but was barely detectable by qRT-PCR analysis, being reduced by a factor of 2^10^ as compared to normal productive infection (data not shown). No viral CP was detected during this period in the plants. Still, the transgene expression, and the resistance reaction against the expressed infectious viral RNA caused a severe stress condition of the plants, manifested by the strong reduction of the plant growth (Figure 1).

When the resistance was broken, at about 1.5–2 months after germination, the plants became fully infected from the endogenous inoculum. The first sign of the resistance break was the appearance of typical patchy mosaic symptoms in the small apical leaves. Also, the viral coat protein was first detected by immunolabeling as small patches in the apical leaf initials, and in the vascular tissue, but not in the apical meristems (Figure 1B). This patchy pattern of the resistance break particularly in the uppermost leaves suggests that it was related to the changing developmental and maturity level of the plants.

Similar, but not identical, virus resistance has been reported in transgenic *N. benthamiana* plants that express the full length genome of *Plum pox potyvirus* (PPV) [68]. In that case the virus resistance occurred only in some of the PPV-expressing transgenic lines, and apparently several different mechanisms contributed to its induction, including RNA silencing.

To identify physiological and molecular processes that are associated with the resistance status of the young plants, we investigated the transcriptome of the transgenic plants before and after resistance break. The microarray analysis revealed multiple gene expression alterations in these plants, and several of them - for example the strong suppression of the translational machinery, enhanced biotic stress responses including the activation of the SAR and HR pathways, hormonal changes or cell division alterations - may all contribute to the resistance.

### Protein translation machinery was strongly suppressed in the BRB transgenic plants

An interesting feature in the transcript profile was the strong down-regulation of the multiple (more than 700) transcripts coding for different components of the translation machinery. The strong reduction of the 40S and 60S ribosomal RNAs, and of other ribosomal genes in the BRB plants, compared to their strong up-regulation in the TMV infected wt plants, and also to their normal expression in the ARB transgenic plants, suggest that the availability of host translational machinery is actively restricted in the BRB transgenic plants. This may directly suppress the accumulation of viral proteins. Furthermore, the reduction of the eIF3 and eEF1A and eEF1B translation initiation and elongation factors, which are known to be needed for the TMV-specific replicase complex [1], [15], [16], [19], [30], [49], [69]–[71], may, to some extent, directly suppress the TMV replication.

Many viruses modify the host translational machinery to increase the viral protein synthesis but not host protein synthesis [55], [72]–[74]. In the case of TMV, the viral genomic and coat protein RNAs are stronger translational templates than host mRNAs [55]. TMV genomic RNA has a long 5′-leader sequence, so called omega sequence, which promotes its translation by efficiently recruiting the 40S and 60S ribosomal subunits to form the 80S-preinitiation complex [74], [75]. The 5′-leader sequence also interacts with the heat shock protein 101 to recruit eIF4F [76]. Thus, TMV should strongly compete for the host cell's translational capacity, even under the situation where the total translation machinery is reduced. Thus the significance of this response, in terms of the induced viral resistance, is not clear.

Many protein degradation pathways and several proteases were induced at the resistant stage but reduced in the TMVi plants, indicating that the BRB transgenic plants may promote the resistance also through enhanced turnover of the viral proteins. The reduced protein synthesis, and enhanced protein degradation were reflected in the reduced soluble protein content, and also in the altered protein profile of the BRB plants.

### TMV-resistant BRB transgenic plants exhibited less photosynthetic damage and higher defense responses than did the ARB transgenic and TMVi plants

Chloroplasts are the main center for many important metabolic functions, and many biotic/abiotic stresses, including virus infections, influence their environment [63]. For instance, TMV infections affects the composition of chloroplast proteins, and efficient TMV accumulation depends on the silencing of the 33k subunit of the OEC and ferridoxin I proteins, which are involved in defense against TMV [51], [62], [64], [77]. Up-regulation of OEC complex proteins in the BRB-TMV plants, and their down-regulation in the the ARB plants may thus relate to the induced defense condition in the BRB-TMV plants.

In the TMVi plants the transcriptome was altered much less than in the transgenic BRB and ARB plants. Interestingly, while induction of defense-related genes is not typical to TMVi plants due to the compatible interaction, this reaction was quite opposite in the BRB and ARB transgenic plants. Induced expression of SAR- and HR-associated proteins in the BRB transgenic plants, and their down-regulation after resistance break or virus infection (ARB and TMVi plants) indicates the incompatible host-virus interactions and induction of the active resistance pathways [61] in the BRB plants. This was also indicated by the expression of the non-functional allele of the N-resistance gene and of other R-genes, observed in the BRB transgenic plants. All these activated defence-related genes and pathways are likely to contribute to the strong TMV resistance condition in the BRB plants.

### Cell division

Several animal studies have revealed that viruses can hijack the host cell division machinery to control the anti-cancer mechanisms [78] and thus provide a suitable environment for virus replication process. One of the cell division check point control protein RAD9A is expressed on very high level (∼140 x) in our ARB transgenic and TMVi plants but not in the BRB transgenic plants. RAD9A protein is known for its high expression levels during high DNA damage conditions. Several cancer studies indicate that cells accumulate RAD9A protein during carcinogenesis and also the RAD9A down-regulation by siRNA reduces the tumorogenesis in the cells [65], [66]. It is not clear how the RAD9A protein increases tumorogenesis, but one hypothesis is that it may induce the expression of adjacent carcinogenic genes. Abnormally high expression of RAD94 in the ARB transgenic and TMVi plants may indicate that it plays some role in the TMV infection process or cell's stress reaction under the virus infection.

Many cyclins (A and B type) were strongly down-regulated in the BRB transgenic plants. Cyclins are involved in the cell cycle regulation by the cyclin dependent protein kinases phosphorylation process (CDKs) [79], [80], and their down-regulation may indicate that the cell cycle is stalled during G2 and M phase in the BRB transgenic plants, leading to their stunted phenotype.

### Conclusions

The microarray analysis reveals that the expression of the infectious TMV genome in the germinating and developing tobacco tissue induces strong alterations in an unique pool of transcripts, many of which may contribute to the TMV resistance status of the plants. One important factor in this condition appears to be the strong enhancement of the SAR- and HR-type defence pathways. Another interesting response is the suppression of the translational machinery, which is a totally unique reaction observed in these plants. This may slow down the synthesis of viral proteins, and also deprive the cells of different host factors (e.g. translation factors eIF-3 and eEF1A and B) which are needed for TMV replication, but the role of these reactions in terms of the virus resistance remains unclear. How all these responses are induced and mutually integrated in the young transgenic plants remains to be solved.

## Methods

### Plant material

The wild type tobacco (*Nicotiana tabacum* cv. xanthi nn) and transgenic tobacco plants, which express the whole TMV genome (strain U1) were grown in greenhouse at 60% relative humidity and in 22°C temperature, with a light/night regime of 16 h light (150 µmol photons m−^2^s−^1^) and 8 h dark. Three replicate samples were collected from the selected transgenic tobacco line, from three BRB plants at 6 weeks, and from the same ARB plants at 8 weeks after germination. At the same time, corresponding sets of the control samples were collected from wild type (wt) plants, which were grown in the same conditions and of the same age as the test plants at the time of sampling. During this collection period all plants remained in the vegetative growth stage. Always the third leaf from the apex (about half of the mature leaf size) was collected, and the collection took place always at the same time of the day (11 am). Wt tobacco plants were mechanically inoculated with TMV at 8 weeks after germination, and samples of systemically infected leaves were collected from these plants at 8 days after inoculation, in parallel with corresponding control samples from healthy wt plants. All the leaf samples were directly frozen in liquid nitrogen and used for RNA extraction. Positive expression of transgene (TMV) RNA was detected in the leaf samples by qRT-PCR prior to the array analysis; TMV RNA was not detectable in these samples by Northern blotting [53] but was detectable on very low level by qRT-PCR. The first set of protein extractions were done from the same samples what were used for the RNA extraction, and additional protein samples were later collected from separate sets of plants of the same line, at the same growth stage and growth conditions.

### RNA extraction, cDNA labeling and microarray hybridization

Total RNA was extracted from healthy wild type (controls), BRB and ARB transgenic, and TMVi wild type tobacco plants by using TRIsure reagent (Bio line, UK) according to manufacturer's instructions. The extracted total RNA was purified with the RNA purification kit (Nucleospin RNA clean-up, Macherey-Nagel) and then subjected to DNaseI treatment (Promega RQ1 RNase free-DNaseI) according to the manufacturer's recommendations. Subsequently, the total RNA was concentrated with Amicon Ultra-0.5 centrifugal filter devices. The cDNA labeling, and Agilent 4×44k microarray hybridization were done according to the manufactures instructions (Center for Biotechnology, Turku, Finland) and the raw numerical data handling and its statistical analysis were done by using Chipster (CSC, Finland) program [81] as previously described [41].

### Annotation of differentially regulated genes in microarray data

The probe information provided by the manufacturer for the Agilent 4×44k microarray was limited and mainly based on EST and cDNA sequences. Therefore, most of this descriptive annotation information was obtained from the http://mapman.gabipd.org/web/guest/mapman-annotationexperts website, with additional information obtained from the websites like JCVI http://plantta.jcvi.org/ and BLAST http://blast.ncbi.nlm.nih.gov/Blast.cgi. The functional grouping of probes was also attained from the mapman.gabipd.org website, with some additional manual adjustments.

### Verification of differentially expressed genes

The microarray results were verified by using the quantitative real-time PCR (RT-qPCR) method by following the MIQE guidelines [82]. A total of 1 µg purified leaf total RNA was used to make cDNA synthesis by using the Revert Aid reverse transcriptase enzyme (product # EPO441, Fermentas) according to the manufacture instructions. The RT-qPCR reactions were performed using 10 ng (3 µl) of diluted cDNA samples (1∶15), gene specific primers (Table S16) and Maxima SYBR Green/Fluorescein RT-qPCR Master Mix (2X) (Product #K0242, Fermentas) with a total volume of 25 µl. For each biological replicate, 3–4 technical replicates were run to reduce the pipetting errors. The standard error of mean was measured from the three biological replicates. The Bio-Rad iQ5 machine was used to perform the RT-qPCR in 96–well plate and the results were calculated by employing the quantification cycle (Cq) method (delta delta Cq). Primer specificity was tested by checking the single peak in the DNA melting curves.

### In situ labeling of the TMV coat protein

Sample sections (7 µm) were prepared from the shoot apical domains of BRB/ARB TMV transgenic plants at 7 weeks after germination, i.e. just at the time of resistance break, by excising and immediate fixing as described previously [83]. The sample sections were initially incubated in phosphate buffered saline (PBS) containing 4% bovine serum albumin at room temperature for 30 min. Later, the samples were subjected to incubation by TMV- specific alkaline phosphatase–conjugated polyclonal antibodies (dilution 1∶50) at 4°C for overnight. Next morning, the samples were washed and stained with freshly prepared fuchsin substrate solution and examined with a Leitz, Laborlux S light microscope (Leica Microsystems AG) at 40× and 100× magnifications.

### Photosynthetic Measurements

Equal amount of leaf samples (1.0 g) from the healthy control, BRB and ARB transgenic plants, and from TMVi tobacco plants were taken and ground in 4 ml of thylakoid isolation buffer (0.3 M sorbitol, 50 mM HEPES/KOH pH 7.4, 5 mM MgCl_2_, 1 mM EDTA, and 1% BSA) with ice cold mortar and pestle. The ground mixture was filtered through the Miracloth and 2 ml of the filtrate was taken and centrifuged at 12000×g for 2 minutes. The chlorophyll concentration measurements were done according to the procedure stated in [84]. The supernatant was removed and the thylakoid pellet was resuspended in oxygen electrode buffer (0.3 M sorbitol, 50 mM HEPES/KOH pH 7.4, 5 mM MgCl_2_ and 1 mM KH_2_PO_4_). The oxygen evolution measurements were carried out by a Clark type electrode by using 0.5 mM DCBQ as electron donor.

### Protein Isolation and 2D gel electrophoresis

Protein extraction for the measurement of the total soluble protein content from the BRB plants and healthy control plants was done from by 0.5 g of leaf tissue, ground inthe ice cold oxygen electrode buffer (0.3 M sorbitol, 50 mM HEPES/KOH pH 7.4, 5 mM MgCl_2_ and 1 mM KH_2_PO_4_). Subsequently, supernantent was isolated and used for protein quantity measurement by using lowry assay.

Protein samples for the electrophoresis analysis from leaves of the healthy control, BRB and ARB transgenic plants and of the TMVi plants were isolated by using TRIsure reagent (Bio line, U.K) according to the manufacture recommendations with some adaptive steps from TRIzol protocol (Invitrogen Inc. USA). The concentrations of isolated protein samples were measured by using Lowry assay. A total of 20 µg protein samples were loaded and run on 1D SDS-page electrophoresis to verify the equal loading of the protein samples. 250 µg of protein samples were taken and mixed with rehydration buffer (8 M urea, 4% CHAPS, 2 M thiourea, 20 mM Tris-HCl, 0.05% bromophenol blue, 100 mM DTT and 5 µl/ml of Bio-lyte ampholyte solution). The protein samples were incubated for 2 hours at room temperature and separated by isoelectric focusing using Bio-Rad 7 cm IPG pH 3–10 strips. Subsequently, the IPG strips were subjected to the second dimension separation with the 15% PAGE gels by using protein II apparatus (Bio-Rad). The protein gels were fixed with isopropanol and acetic acid treatments for 15 minutes each and incubated overnight with coomassie blue stain (Page Blue staining kit, Fermentas). In the next morning, gels were destained and photographed. Optionally, the gels were stained again with silver staining kit (Page silver staining kit, Fermentas) according to manufacturer's instructions to analyze even the low abundance proteins.

### ELISA analysis of the virus titers

Different viruses used in this study (PVY, PVX, PVA and TMV) were detected by using double antibody sandwich enzyme-linked immunosorbent assay (DAS-ELISA) according to the manufacturers guidelines (Bioreba, Reinach, Switzerland) with slightly modified protocol. The commercial polyclonal, alkaline phosphatases conjugated antibodies (Bioreba, Reinach, Switzerland) against all viruses were diluted to 1∶10000 for use. The ELISA reactions were developed by using the p-Nitrophenyl phosphate as a substrate, and measured at 405 nm absorbance by using the ELISA plate reader (Benchmark, Bio-Rad, Hercules, CA, U.S.A.). 100 ng of purified virions of the corresponding viruses were used as internal standards.

## Supporting Information

Figure S1 **Graphic presentation of the altered protein synthesis-related transcripts (log2 value>1), as detected and portrayed by the MapMan software from the microarray data of the BRB-, ARB-TMV transgenic and TMVi plants.** The blue- and red squares and bars represent the up- and down-regulated transcripts, respectively, and the picture frame depicts their functional location in the cell. The boxes portrayed inside the nuclear circles indicate the alterations in the transcription- and mRNA processing-related transcripts, respectively. Alterations of the translation-related transcripts are portrayed in the cytoplasm, separately for the plastidic, mitochondrial and cytoplasmic ribosomes.
(TIF)

Figure S2 **Graphic presentation of the altered stress-related transcripts (log2 value>1) as detected and portrayed by using the MapMan software from the microarray data of the BRB- and ARB-TMV transgenic and TMVi plants.** The blue and red squares represent the up- and down-regulated transcripts, respectively, that are involved in the different signaling pathways and in different stress responces.
(TIF)

Figure S3 **1D-SDS-PAGE gels (left panel), and 2D-polyacrylamide gel electrophoresis (2D-PAGE) (right panel) showing, respectively, the equal loading of samples and the levels of various individual proteins.** Upper two panels show BRB-TMV transgenic plants protein samples analysis at before resistance break stage, the middle two panels show the ARB-TMV transgenic plants protein samples analysis at after resistance break stage, and the bottom two panels show samples from the TMV-infected wild type tobacco plants (TMVi). All gels were stained with coomassie blue. Molecular weight ladder (Thermo Scientific) contains markers for the 250, 130, 100, 70, 55, 35, 25, 15, and 10 kDa proteins – the most important ones are marked in the panels. The TMV CP is indicated with the * in the ARB and TMVi gels.
(TIF)

Table S1 **Normalized microarray data showing both down- (sheet1) and up-regulated (sheet 2) transcripts in the leaves of BRB-TMV transgenic tobacco plants.**
(XLSX)

Table S2 **Normalized microarray data showing both down- (sheet1) and up-regulated (sheet 2) transcripts in the leaves of ARB-TMV transgenic tobacco plants.**
(XLSX)

Table S3 **Normalized microarray data showing both down- (sheet1) and up-regulated (sheet 2) transcripts in the leaves of TMVi wild type tobacco plants.**
(XLS)

Table S4 **Down-regulated transcripts related to the protein synthesis, degradation and amino acid metabolism in the leaves of BRB-, ARB- transgenic and TMVi plants.**
(DOCX)

Table S5 **Protein synthesis, degradation and amino acid metabolism related up-regulated transcripts detected in the leaves of BRB-, ARB- transgenic and TMVi plants.**
(DOCX)

Table S6 **Biotic stress related down-regulated transcripts detected in the leaves of BRB-, ARB- transgenic and TMVi plants.**
(DOCX)

Table S7 **Biotic stress related up-regulated transcripts detected in the leaves of BRB-, ARB- transgenic and TMVi plants.**
(DOCX)

Table S8 **Hormones and development related down-regulated detected in the leaves of BRB-, ARB- transgenic and TMVi plants.**
(DOCX)

Table S9 **Hormones and development related up-regulated detected in the leaves of BRB-, ARB- transgenic and TMVi plants.**
(DOCX)

Table S10 **Photosynthesis and carbohydrate metabolism related down-regulated transcripts detected in the leaves of BRB-, ARB- transgenic and TMVi plants.**
(DOCX)

Table S11 **Photosynthesis and carbohydrate metabolism related up-regulated transcripts detected in the leaves of BRB-, ARB- transgenic and TMVi plants.**
(DOCX)

Table S12 **Cell division and DNA-binding related down-regulated transcripts detected in the leaves of BRB-, ARB- transgenic and TMVi plants.**
(DOCX)

Table S13 **Cell division and DNA-binding related up-regulated transcripts detected in the leaves of BRB-, ARB- transgenic and TMVi plants.**
(DOCX)

Table S14 **Different up-regulated transcripts in the BRB-TMV transgenic plants after subtracting the up-regulated transcripts from other ARB-TMV, TMVi and different VRS expressing transgenic tobacco plants (HcPro, AC2 and P25).**
(DOCX)

Table S15 **Different down-regulated transcripts in the BRB-TMV transgenic plants after subtracting the up-regulated transcripts from other ARB-TMV, TMVi and different VRS expressing transgenic tobacco plants (HcPro, AC2 and P25).**
(DOCX)

Table S16 **Primers used in RT-qPCR experiment for validation of microarray data.**
(DOCX)
