# Supplementary figures and images for: Multiple Different Defense Mechanisms Are Activated in the Young Transgenic Tobacco Plants Which Express the Full Length Genome of the Tobacco Mosaic Virus, and Are Resistant against this Virus
Source: PLoS One. 2014 Sep 22;9(9):e107778. doi: 10.1371/journal.pone.0107778 (PMC4171492; doi:10.1371/journal.pone.0107778)

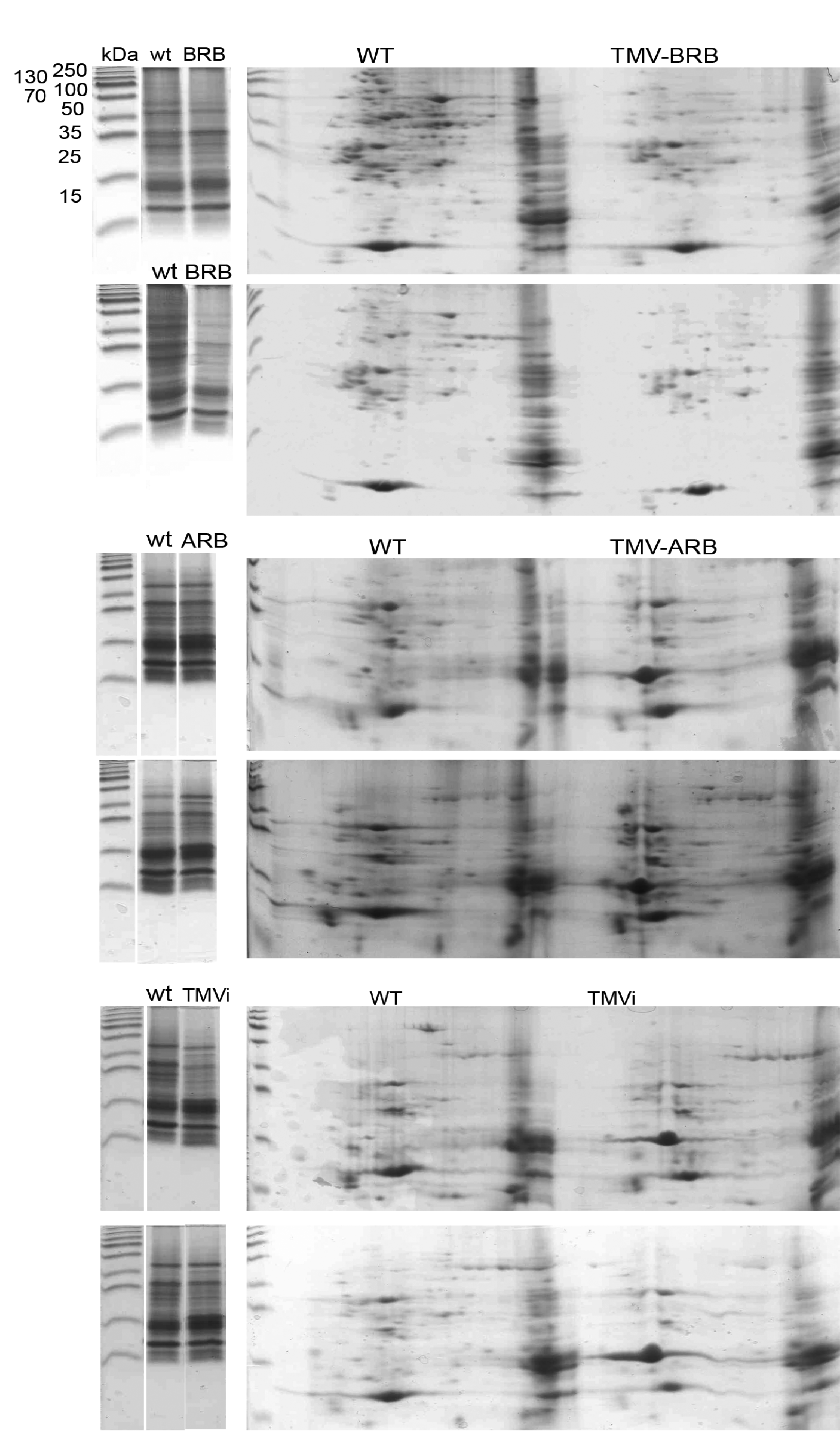

Supplement: Figure S3 — 1D-SDS-PAGE gels (left panel), and 2D-polyacrylamide gel electrophoresis (2D-PAGE) (right panel) showing, respectively, the equal loading of samples and the levels of various individual proteins. Upper two panels show BRB-TMV transgenic plants protein samples analysis at before resistance break stage, the middle two panels show the ARB-TMV transgenic plants protein samples analysis at after resistance break stage, and the bottom two panels show samples from the TMV-infected wild type tobacco plants (TMVi). All gels were stained with coomassie blue. Molecular weight ladder (Thermo Scientific) contains markers for the 250, 130, 100, 70, 55, 35, 25, 15, and 10 kDa proteins – the most important ones are marked in the panels. The TMV CP is indicated with the * in the ARB and TMVi gels. (TIF) [file pone.0107778.s003.tif]
