# Supplementary material for: Multiple Different Defense Mechanisms Are Activated in the Young Transgenic Tobacco Plants Which Express the Full Length Genome of the Tobacco Mosaic Virus, and Are Resistant against this Virus
Source: PLoS One. 2014 Sep 22;9(9):e107778. doi: 10.1371/journal.pone.0107778 (PMC4171492; doi:10.1371/journal.pone.0107778)
Supplement: Table S4 — Down-regulated transcripts related to the protein synthesis, degradation and amino acid metabolism in the leaves of BRB-, ARB- transgenic and TMVi plants. (DOCX) [file pone.0107778.s007.docx]

| **Table S4. A list of down-regulated genes related to protein-synthesis, post-translational modifications and degradation, and amino acid synthesis and degradation in the BRB-, ARB-TMV transgenic and in TMVi plants.** | | |
| --- | --- | --- |
|  | **Total number of positive detections** | **Range of fold -change enhancement** |
| **BRB-TMV TRANSGENIC PLANTS** | | |
| **Protein synthesis, degradation and amino acid related** | **750** |  |
| 40S ribosomal proteins, various | 222 | 0.22-0.5 x |
| 60S ribosomal proteins, various | 391 | 0.18-0.49 x |
| Elongation: Nascent polypeptide complex, LOS1 and elongation factors | 18 | 0.28-0.49 x |
| Translation initiation: EIF2 gamma, eIF-4A1 and eIF-3 | 15 | 0.33-0.49 x |
| Protein degradation: ubiquitin, various proteases | 21 | 0.27-0.49 x |
| Protein folding: chaperons, various and GrpE proteins | 19 | 0.26-0.48 x |
| Post translation related, various | 5 | 0.21-0.47 x |
| Organellar ribosomal proteins : various chloroplast and other unknown organelle | 8 | 0.26-0.49 x |
| ribosomal RNA: 18S and ribosome biogenesis regulator like | 7 | 0.21-0.49 x |
| Protein targeting: Importin,Tic40 and TOC75 complexes | 5 | 0.35-0.49 x |
| aromatic amino acid biosynthesis related, various | 7 | 0.27-0.49 x |
| Methionine biosynthesis related, various | 7 | 0.39-0.49 x |
| Branched amino acid synthesis related, various | 7 | 0.40-048 x |
| Amino acid related, various | 10 | 0.24-0.49 x |
| Miscellaneous | 8 | 0.36-0.47 x |
| **ARB-TMV TRANSGENIC PLANTS** | | |
| **Protein synthesis, degradation and amino acid related** | **120** |  |
| [4Fe-4S] cluster assembly factor | 2 | 0.39-0.4 x |
| ATP-dependent protease | 3 | 0.39-0.46 x |
| BTB/POZ and TAZ domain-containing protein | 3 | 0.17-0.33 x |
| Eukaryotic translation initiation factor 3 subunit A | 2 | 0.34-0.39 x |
| RNA transcription: Sigma factor and DNA polymerases related | 6 | 0.23-0.36 x |
| F-box family protein, various | 5 | 0.26-0.49 x |
| FtsH protease related | 5 | 0.35-0.49 x |
| Nodulation receptor kinase related | 4 | 0.25-0.47 x |
| Peptidase: M48, S41 and M50 family related | 4 | 0.06-0.18 x |
| Peroxisomal membrane protein | 3 | 0.2-0.4 x |
| Phosphoenolpyruvate carboxylase kinase related | 4 | 0.14-0.35 x |
| Phosphatase : 2a and 2c | 3 | 0.24-0.46 x |
| Proline oxidase | 3 | 0.2-0.26 x |
| Ribosomal protein: L7Ae like | 4 | 0.45-0.49 x |
| C3HC4 and H2 RING finger protein related | 8 | 0.29-0.43 x |
| FKF1 related | 2 | 0.38-0.41 x |
| Serine protease and carboxypeptidases-like protein | 7 | 0.16-0.49 x |
| Subtilisin-like protease | 4 | 0.33-0.5 x |
| Ubiquitin related, various | 4 | 0.18-0.49 x |
| Vacuolar processing enzyme-1a | 3 | 0.33-0.44 x |
| Zinc metalloprotease | 2 | 0.42 x |
| Proteases, various | 14 | 0.06- 0.4 x |
| Post transnational Modifications, various | 12 | 0.07-0.5 x |
| Miscellaneous | 13 | 0.23-0.49 x |
| **TMVi PLANTS** | | |
| **Protein synthesis, degradation and amino acid related** | **44** |  |
| Ribosomes related: various types of 30S, unknown ribosomal proteins and 26S ribosomal RNA | 7 | 0.4 - 0.49 x |
| Proteases and peptidases: FtsH, M50 peptidase, ATP dependent peptidase, metallo protease and serine type | 13 | 0.26- 0.49 x |
| Ubiquitin, various | 6 | 0.32- 0.49 x |
| Post translational: CBL interacting kinase 23, protein kinases and signal transducers | 11 | 0.37- 0.49 x |
| Miscellaneous | 7 | 0.34- 0.49 x |
