# Supplementary material for: Multiple Different Defense Mechanisms Are Activated in the Young Transgenic Tobacco Plants Which Express the Full Length Genome of the Tobacco Mosaic Virus, and Are Resistant against this Virus
Source: PLoS One. 2014 Sep 22;9(9):e107778. doi: 10.1371/journal.pone.0107778 (PMC4171492; doi:10.1371/journal.pone.0107778)
Supplement: Table S5 — Protein synthesis, degradation and amino acid metabolism related up-regulated transcripts detected in the leaves of BRB-, ARB- transgenic and TMVi plants. (DOCX) [file pone.0107778.s008.docx]

| **Table S5. A list of up-regulated genes related to protein-synthesis, post-translational modifications and degradation, and amino acid synthesis and degradation in the BRB-, ARB-TMV transgenic and in TMVi plants.** | | |
| --- | --- | --- |
|  | **Total number of positive detections** | **Range of fold -change enhancement** |
| **BRB- TMV TRANSGENIC PLANTS** | | |
| **Protein synthesis, degradation and amino acid related** | **150** |  |
| Ubiquitin related : conjugating, ligase E3 and polyubiquitins | 22 | 2- 4.3 x |
| Carboxylate peptidases: serine and pyrrolidine type | 9 | 2- 3.8 x |
| Autophagy 8c protein | 5 | 2.1-13.5 x |
| Cathepsin B-like cysteine protease | 5 | 2.1- 4 x |
| Subtilase related | 5 | 2- 3.8 x |
| RING zinc finger protein-like protein | 5 | 2- 4.2 x |
| F-box family proteins | 9 | 2.1- 4.6 x |
| CBL interacting protein kinase | 4 | 2- 2.2 x |
| Serine / threonine protein kinases | 11 | 2 -7.2 x |
| Translation initiation factor: SUI1 and eIF-4A1 | 6 | 2- 5.9 x |
| Ribosomal proteins: 30S, 60S and 18S | 6 | 2- 6.3 x |
| RNA polymerase sigma subunit SigD | 4 | 5.3–5.7 x |
| Urease accessory protein G | 4 | 2-3.1 x |
| Glutamate decarboxylase | 4 | 3.3–4.4 x |
| Proteases: cysteine, aspartate, cucmisin and FtsH type | 8 | 2- 6.4 x |
| Peptidases: M48, M50, Xaa-Pro | 5 | 2.1-3.9 x |
| Protein Kinases: Mapk, LysM-domain and CIP 1 like | 8 | 2.1-3 x |
| Posttranslational : Pentatricopeptide (PPR) repeats, OBP3-responsive gene 1 and TLP1 | 7 | 2.6- 7.1 x |
| Protein targeting: ER, Peroxisomes and vacuoles | 4 | 2.2-2.6 x |
| Amino acid synthesis, various | 8 | 2- 3.4 x |
| Protease inhibitors, various | 4 | 2- 6.1 x |
| Miscellaneous | 7 | 2.1-8 x |
| **ARB-TMV TRANSGENIC PLANTS** | | |
| **Protein synthesis, degradation and amino acid related** | **135** |  |
| Cysteine, serine and aspartic- proteases, protease inhibitors and carboxypeptidases related | 16 | 2- 21.6 x |
| Metallocarboxypeptidase inhibitor IIa and Metallothionein protein like | 6 | 2-26 x |
| Translation initiation, poly a binding and ribosomal (60S and 30S ) proteins related | 13 | 2-23.2 x |
| Ubiquitin- associated ligase, E2 and E3 ring like proteins. FKF family, skp1 and polyubiquitin like proteins | 29 | 2- 5.1 x |
| Arginine and ornithine decarboxylase related | 9 | 4.3-20.2 x |
| Aromatic, asparagine, serine, proline, methionine, alanine and asparagine amino acid biosynthesis related | 25 | 2- 8.6 x |
| Nucleus, peroxisomes and secretory pathway targeting proteins related | 6 | 2.1-5.2 x |
| Leucin, lysine and threonine amino acid degradation related | 7 | 2.6-4.5 x |
| protein disulfide isomerases | 3 | 4.9-5.8 x |
| Branched chain amino acid related protein | 1 | 2.1 x |
| AAA-type ATPase and subtilase family protein related | 6 | 2.1-2.9 x |
| Phosphatase 2c protein related | 3 | 2.8-3.1 x |
| Nek4, SNF-1 kinases related | 2 | 2.8-2.9 x |
| Miscellaneous | 9 | 2-39.3 x |
| **TMVi PLANTS** | | |
| **Protein synthesis, degradation and amino acid related** | **197** |  |
| 60 S type ribosomal proteins; L 5-10,10 A,11,13, 13 A,14,15,17-26,28,30,34,37, 38, 41 and 44 subunits | 114 | 2- 23.2 x |
| 40 S type ribosomal proteins: S 3,4,8, 10, 12, 14, 15,17,19, 20, 23-27 sub units | 55 | 2 - 4.2 x |
| Ubiquitin associated and extensin protein related | 11 | 2- 3.6 x |
| Posttranslational modification: protein phosphatases | 5 | 2.2- 5.2 x |
| Protein folding related | 2 | 2.1– 2.2 x |
| Elongation factor: 2 and 5A | 2 | 2-2.3 x |
| Nucleus targeting: ABI five binding protein 3 | 2 | 2.4-2.5 |
| Amino acid degradation: proline dehydrogenase and 4-alpha hydroxytetrahydrobiopterin dehydratase | 3 | 2.7-3.5 x |
| Phosphatidylinositol 3- and 4-kinase | 1 | 27.7 x |
| Miscellaneous | 2 | 2-2.7 x |
