# Supplementary material for: Multiple Different Defense Mechanisms Are Activated in the Young Transgenic Tobacco Plants Which Express the Full Length Genome of the Tobacco Mosaic Virus, and Are Resistant against this Virus
Source: PLoS One. 2014 Sep 22;9(9):e107778. doi: 10.1371/journal.pone.0107778 (PMC4171492; doi:10.1371/journal.pone.0107778)
Supplement: Table S6 — Biotic stress related down-regulated transcripts detected in the leaves of BRB-, ARB- transgenic and TMVi plants. (DOCX) [file pone.0107778.s009.docx]

| **Table S6. A list of down-regulated genes related to different stress responses in the BRB-, ARB-TMV transgenic and in TMVi plants.** | | |
| --- | --- | --- |
|  | **Total number of positive detections** | **Range of fold -change enhancement** |
| **BRB-TMV TRANSGENIC PLANTS** | | |
| **BIOTIC AND ABIOTIC STRESS RELATED** | **59** |  |
| Heat shock proteins: DnaJ family,17.8 kDa and chaperons | 17 | 0.24- 0.48 x |
| Arabinogalactan protein | 5 | 0.41-0.46 x |
| Thaumatin-like protein | 2 | 0.39-0.47 x |
| Phosphosulfolactate synthase-related protein | 3 | 0.39-0.47 x |
| ATP binding | 6 | 0.23-0.48 x |
| Snakin-1 (SN1) gene | 2 | 0.41-0.48 x |
| UP-9A related | 3 | 0.37-0.38 x |
| Defense related: elicitors, thionins, PR4 and osmotin like proteins | 5 | 0.42-0.49 x |
| Miscellaneous | 16 | 0.15-0.49 x |
| **ROS and oxidoreductase related** | **17** |  |
| Cytochrome P450, various | 8 | 0.19-0.48 x |
| Peroxidase: Ascorbate peroxidase 1 and membrane related | 5 | 0.25-0.44 x |
| Peroxiredoxin protein | 3 | 0.36-0.46 x |
| Glucose-methanol-choline (gmc) oxidoreductase | 1 | 0.45 x |
| **ARB-TMV TRANSGENIC PLANTS** | | |
| **BIOTIC AND ABIOTIC STRESS RELATED** | **86** |  |
| Heat shock proteins: DnaJ family,17.8 kDa and chaperons | 21 | 0.09- 0.49 x |
| Wound induce proteins related | 5 | 0.22 -0.47 x |
| SAR8.2b protein related | 6 | 0.25-0.41 x |
| Elicitor inducible protein | 9 | 0.03-0.49 x |
| Low temperature and salt responsive protein | 5 | 0.3-0.45 x |
| Methanol inducible protein (MIG-21) | 5 | 0.35-0.39 x |
| Rhodanese-like domain-containing protein | 3 | 0.27-03 x |
| Aluminum-induced protein | 2 | 0.44-0.48 x |
| HR proteins: Band 7 family and HR induced proteins | 3 | 0.44-0.46 x |
| Germin-like protein | 2 | 0.3-0.35 x |
| NDR1-like protein | 2 | 0.44-0.48 x |
| Sam2B protein | 2 | 0.32-0.35 x |
| CAPIP1-like related | 2 | 0.22-0.43 x |
| Miscellaneous | 19 | 0.23-0.49 x |
| **ROS and oxidoreductase related** | **45** |  |
| Alcohol oxidase | 3 | 0.27-0.45 x |
| Catalase related | 4 | 0.4-0.49 x |
| Cytochrome p450 related | 12 | 0.06-0.47 x |
| Glutathione S-transferase | 6 | 0.11-0.37 x |
| Superoxide dismutase | 3 | 0.38-0.40 x |
| Thioredoxin proteins related | 10 | 0.2-0.48 x |
| Glutaredoxin and monothiol glutaredoxin | 2 | 0.38-0.46 x |
| Miscellaneous | 5 | 0.15-0.43 x |
| **TMVi PLANTS** | | |
| **BIOTIC AND ABIOTIC STRESS RELATED** | **32** |  |
| Heat shock proteins, various | 10 | 0.33- 0.49 x |
| Chitinases: acidic and basic and endochitinases | 3 | 0.22- 0.43 x |
| Pollen coat-like proteins | 2 | 0.08 x |
| Wound-induced protein | 5 | 0.31- 0.49 x |
| Cold acclimation related proteins | 3 | 0.44- 0.48 x |
| Short chain alcohol dehydrogenase | 3 | 0.29- 0.48 x |
| Alcohol oxidase-related | 1 | 0.42 x |
| Miscellaneous | 5 | 0.32- 0.49 x |
| **ROS and oxidoreductase related** | **14** |  |
| Glutathione s-transferase related | 5 | 0.3- 0.41 x |
| Peroxidases related | 4 | 0.46- 0.5 x |
| Cytochrome 450: monooxygenase | 4 | 0.25- 0.45 x |
| Glutathione peroxidase | 1 | 0.3 x |
