# Supplementary material for: Multiple Different Defense Mechanisms Are Activated in the Young Transgenic Tobacco Plants Which Express the Full Length Genome of the Tobacco Mosaic Virus, and Are Resistant against this Virus
Source: PLoS One. 2014 Sep 22;9(9):e107778. doi: 10.1371/journal.pone.0107778 (PMC4171492; doi:10.1371/journal.pone.0107778)
Supplement: Table S7 — Biotic stress related up-regulated transcripts detected in the leaves of BRB-, ARB- transgenic and TMVi plants. (DOCX) [file pone.0107778.s010.docx]

| **Table S7. A list of the up-regulated genes related to different stress responses in the BRB-, ARB-TMV transgenic and in TMVi plants.** | | |
| --- | --- | --- |
|  | **Total number of positive detections** | **Range of fold -change enhancement** |
| **BRB-TMV TRANSGENIC PLANTS** | | |
| **BIOTIC AND ABIOTIC STRESS RELATED** | **174** |  |
| Glycine-rich protein precursor | 13 | 3- 4.8 x |
| Osmotin precursor | 8 | 2.9-14.3 x |
| Cold acclimation protein | 8 | 2.6- 9.4 x |
| Dehydrin-like protein | 8 | 2.1-3.2 x |
| Heat shock proteins: DnaJ 20, chaperons and HSP proteins | 20 | 2.2 -24.1 x |
| Wound-responsive and tumor related proteins | 16 | 2.6-10 x |
| Chitinases: acidic and basic and endochitinases | 17 | 2 -10.6 x |
| Elicitor inducible proteins | 16 | 2- 10.9 x |
| SAR 8.2 proteins: b,c,d,e and m | 13 | 2.1-3.2 x |
| Pathogenesis-related protein (PRP) 1 | 7 | 2-3.4 x |
| HR related and apoptosis inhibitor proteins | 12 | 2-2.7 x |
| Disease resistance protein (TIR-NBS-LRR class) and LRR domain proteins | 6 | 2- 3 x |
| Defensins, jacalin lectin and thaumatin related proteins | 6 | 2 - 4 x |
| Abscisic acid related | 3 | 2-2.1 x |
| Phytoalexins related | 2 | 2.4- 4 x |
| Pollen coat like proteins | 2 | 2.9-3.1 x |
| Xenobiotics degradation related | 2 | 2 x |
| Methanol inducible proteins | 4 | 2.1- 4.4 x |
| Jasmonic acid 2 | 1 | 2.2 x |
| VAMP protein SEC22 | 2 | 2.5 x |
| Miscellaneous | 8 | 2.6- 6.7 x |
| **ROS, Peroxidase and glutathione transferase related** | **73** |  |
| Peroxidases: secretory, cationic and glutathionic related | 21 | 2- 9.3 x |
| Cytochromes P450 related, various | 19 | 2-13.9 x |
| Redox: heme, thioredoxins and glutaredoxins related | 7 | 2-5.6 x |
| Glutathione transferase related | 18 | 2-9 x |
| Catalase related | 6 | 2.5- 2.8 x |
| Membrane steroid binding protein 1 | 2 | 2.2-2.4 x |
| **ARB-TMV TRANSGENIC PLANTS** | | |
| **BIOTIC AND ABIOTIC STRESS RELATED** | **152** |  |
| Cell death associated proteins | 2 | 2.2-7.1 x |
| Chitinases (acidic, basic and endochitinases) | 12 | 2.3 – 7.3 x |
| DBP1 interacting proteins 3 | 3 | 8.6-17.6 x |
| Carbohydrate oxidase related | 3 | 2.9- 24.1 x |
| Defensins, Thioinin, Fibroid FB2, Thaumatin, Germin related proteins | 23 | 2 – 47.9 x |
| Dehydration response proteins | 8 | 2.3-6 x |
| Miscellaneous | 22 | 2-8.6 x |
| Pathogenesis related proteins, 1a, 1b, 4A, 4B, R, | 17 | 2.2 – 6.6 x |
| Disease resistance proteins | 3 | 2.1 -2.4 x |
| Elicitor inducible proteins related | 2 | 2.2-2.3 x |
| SAR-related proteins |  |  |
| TOM1multiplication protein and TMV viral envelope protein | 2 | 3.5-1408 x |
| Hairpin inducing proteins | 3 | 2- 2.2 x |
| Heat shock proteins (including class I,III) | 25 | 2.0-8.1 x |
| Xenobiotics degradation related | 2 | 2.1-2.8 x |
| Polyphenol oxidase and reticuline oxidase related | 6 | 2.1-10.4 x |
| Wound induced and tumor related proteins | 3 | 2- 15.5 x |
| Universal stress family proteins | 2 | 2-2.1 x |
| Trypsin, kunitz, latex serine and lemir protease inhibitors related | 10 | 2.2- 45.3 x |
| Senescence-associated protein related | 2 | 3.9-4.8 x |
| sn-1 related protein | 2 | 2.7-3.6 x |
| **ROS, Peroxidase and glutathione transferase related** | **43** |  |
| Peroxidases: secretory, cationic, glutathionic and thioredoxins related | 18 | 2- 6.6 x |
| Cytochromes P450 related, various | 10 | 2-7.6 x |
| Redox: heme, thioredoxins and glutaredoxins related | 11 | 2-5 x |
| Glutathione transferase related | 3 | 3.9-4.2 x |
| Nitric oxide reductase related | 1 | 3.4 x |
| **TMVi PLANTS** | | |
| **BIOTIC AND ABIOTIC STRESS RELATED** | **41** |  |
| Heat shock proteins, various | 12 | 2 - 4.0 x |
| Chitinases: acidic and basic and endochitinases | 9 | 2.2 - 3.4 x |
| Pathogenesis-related protein (PRP) 1 | 11 | 2.1- 11.6 x |
| Senescence-associated protein | 2 | 3.1- 3.8 x |
| SAR 8.2 protein | 1 | 3.3 x |
| Viral envelope protein | 1 | 1421 x |
| Miscellaneous | 5 | 2.2-3.3 x |
| **ROS and oxidoreductase related** | **20** |  |
| Oxidoreductase: Nitric- oxide, disulfide and copper-flavone reductases | 5 | 2.2-3.8 x |
| Glutathione s-transferase related | 4 | 2.1- 12.6 x |
| Peroxidases related | 4 | 2- 4.6 x |
| Cytochrome 450: monooxygenase | 4 | 2- 3.5 x |
| GDP-mannose 3,5-epimerase | 2 | 2-2.3 x |
| Redox: thioredoxins related | 1 | 4.2 x |
