# Supplementary material for: Multiple Different Defense Mechanisms Are Activated in the Young Transgenic Tobacco Plants Which Express the Full Length Genome of the Tobacco Mosaic Virus, and Are Resistant against this Virus
Source: PLoS One. 2014 Sep 22;9(9):e107778. doi: 10.1371/journal.pone.0107778 (PMC4171492; doi:10.1371/journal.pone.0107778)
Supplement: Table S8 — Hormones and development related down-regulated detected in the leaves of BRB-, ARB- transgenic and TMVi plants. (DOCX) [file pone.0107778.s011.docx]

| **Table S8. A list of down-regulated genes related to hormones and development in the BRB-, ARB-transgenic and in TMVi plants.** | | |
| --- | --- | --- |
|  | **Total number of positive detections** | **Range of fold -change enhancement** |
| **BRB-TMV TRANSGENIC PLANTS** | | |
| **Hormones and development related** | **23** |  |
| WD-40 repeat protein | 7 | 0.24-0.49 x |
| Dem protein-related | 2 | 0.41-0.45 x |
| Pale cress (PAC) protein | 2 | 0.29-0.39 x |
| ARF and phytochrome kinase related | 2 | 0.32-0.33 x |
| Proto dermal factor | 1 | 0.08 x |
| Miscellaneous | 9 | 0.21-0.48 x |
| **ARB-TMV TRANSGENIC PLANTS** | | |
| **Hormones and development related** | **59** |  |
| 1-aminocyclopropane-1-carboxylate oxidase | 7 | 0.14-0.49 x |
| AP2 domain containing proteins | 2 | 0.26-0.39 x |
| Auxin: SAUR, transporter, Stem-specific protein TSJT1, Amino acid permease and responsive proteins | 8 | 0.16-0.45 x |
| 9-cis-epoxycarotenoid dioxygenase | 2 | 0.06-0.07 x |
| Abscisic acid: FIP1, Short-chain dehydrogenase/reductase (SDR) and Zeaxanthin epoxidase | 5 | 0.11-0.49 x |
| Gibberellin 20-oxidase | 3 | 0.14-0.37 x |
| SAM-sterol-C-methyltransferase | 1 | 0.48 x |
| 3-beta hydroxysteroid dehydrogenase/isomerase | 1 | 0.36 x |
| Auxin associated family protein | 3 | 0.38-0.44 x |
| Auxin repressed/dormancy associated protein | 4 | 0.21-0.46 x |
| LEA proteins related | 6 | 0.14-0.25 x |
| Pentatricopeptide (PPR) repeat-containing protein | 6 | 0.18-0.46 x |
| Senescence related | 4 | 0.13-0.42 x |
| Circidian clock: Early flowering 4 and Plastid transcriptionally active 16 | 2 | 0.38-0.44 x |
| Miscellaneous: Embryo defective, Gigantea protein, embryo arrest and lipoxygense related | 5 | 0.16-0.43 x |
| **TMVi PLANTS** | | |
| **Hormones and development related** | **33** |  |
| Ethylene signal transduction: ERF5 and ethylene receptor related | 5 | 0.29- 0.48 x |
| Gibberellin oxidase-like protein related | 4 | 0.32- 0.43 x |
| Lipoxygenases | 2 | 0.35- 0.43 x |
| ACC oxidase related | 2 | 0.29- 0.33 x |
| ABA: Carotenoid cleavage dioxygenase 1 and  Short-chain dehydrogenase/reductase | 2 | 0.4- 0.5 x |
| Auxin repressed protein | 1 | 0.48 x |
| Dormancy associated protein | 3 | 0.38- 0.44 x |
| Senescence associated gene 21 and DIN 1 related | 7 | 0.22- 0.44 x |
| Late embryogenesis abundant protein Lea5 | 3 | 0.35- 0.43 x |
| Miscellaneous | 4 | 0.42- 0.49 x |
