# Supplementary material for: Multiple Different Defense Mechanisms Are Activated in the Young Transgenic Tobacco Plants Which Express the Full Length Genome of the Tobacco Mosaic Virus, and Are Resistant against this Virus
Source: PLoS One. 2014 Sep 22;9(9):e107778. doi: 10.1371/journal.pone.0107778 (PMC4171492; doi:10.1371/journal.pone.0107778)
Supplement: Table S9 — Hormones and development related up-regulated detected in the leaves of BRB-, ARB- transgenic and TMVi plants. (DOCX) [file pone.0107778.s012.docx]

| **Table S9. A list of up-regulated genes related to hormones and development in the BRB-, ARB-TMV transgenic and in TMVi plants.** | | |
| --- | --- | --- |
|  | **Total number of positive detections** | **Range of fold -change enhancement** |
| **BRB-TMV TRANSGENIC PLANTS** | | |
| **Hormones and development related** | **80** |  |
| Ethylene signaling related | 10 | 2- 8.1 x |
| Auxin responsive and dormancy associated | 14 | 2-15.5 x |
| Ethylene synthesis related | 10 | 2-4 x |
| Senescence-associated protein | 11 | 2.2-9 x |
| Auxin associated and repressed proteins, various | 6 | 10.8-15.2 x |
| Abscisic acid synthesis related | 8 | 2-3.9 x |
| LEA proteins | 4 | 2-2.4 x |
| Nodulin family protein | 5 | 2.3-3.5 x |
| Tetraspanin 8 family protein | 3 | 2-2.4 x |
| BYPASS 1 protein | 2 | 2 x |
| development related, various | 6 | 2.1-3 x |
| Gibberellin-responsive protein | 1 | 5.1 x |
| **ARB-TMV TRANSGENIC PLANTS** | | |
| **Hormones and development related** | **61** |  |
| Benzodiazepine receptor related | 2 | 3.6-4.1 x |
| Auxin responsive family related | 3 | 2.3-3.8 x |
| Ethylene related, various | 10 | 3.3-4.4 x |
| Snakin 2, Gibberellin receptor related | 7 | 2- 6.6 x |
| Jasmonic acid, various | 8 | 2.2-26 x |
| Abscisic acid related, various | 4 | 2.8-3.3 x |
| LEA proteins, embryo defective and endosperm related | 4 | 2- 4.1 x |
| Patatin 2 | 2 | 2- 35 x |
| Embryo abundant and specific proteins related | 6 | 2.8-4.5 x |
| Senescence associated protein | 5 | 2-5.2 x |
| WD repeat protein | 3 | 2.1-2.4 x |
| SCARECROW gene regulator | 1 | 2.2 x |
| Jasmonic acid 2 | 1 | 2.5 x |
| En/Spm-like transposon protein | 1 | 11.2 x |
| Miscellaneous: CAXX, CEN1, GhHOX1 and mtN3 protein related | 4 | 2- 20.8 x |
| **TMVi PLANTS** | | |
| **Hormones and development related** | **8** |  |
| Ethylene: Multiprotein bridging factor 1C and 2-oxoglutarate- dependent di oxygenase | 3 | 2-13 x |
| Auxin responsive related | 1 | 2. 25 x |
| Development: Protodermal factor 1 and pale cross related | 2 | 2.2- 3.9 x |
| Miscellaneous | 2 | 2- 2.3 x |
