# Supplementary material for: Multiple Different Defense Mechanisms Are Activated in the Young Transgenic Tobacco Plants Which Express the Full Length Genome of the Tobacco Mosaic Virus, and Are Resistant against this Virus
Source: PLoS One. 2014 Sep 22;9(9):e107778. doi: 10.1371/journal.pone.0107778 (PMC4171492; doi:10.1371/journal.pone.0107778)
Supplement: Table S10 — Photosynthesis and carbohydrate metabolism related down-regulated transcripts detected in the leaves of BRB-, ARB- transgenic and TMVi plants. (DOCX) [file pone.0107778.s013.docx]

| **Table S10. A list of down-regulated genes related to photosynthesis and carbohydrate metabolism in the BRB-, ARB-TMV transgenic and in TMVi plants.** | | |
| --- | --- | --- |
|  | **Total number of positive detections** | **Range of fold -change enhancement** |
| **BRB-TMV TRANSGENIC PLANTS** | | |
| **Photosynthesis and carbohydrate metabolism related** | **41** |  |
| NADP-dependent glyceraldehyde-3-phosphate dehydrogenase | 3 | 0.20-0.22 x |
| Ribulose bisphosphate carboxylase activase | 3 | 0.34-0.40 x |
| Ferredoxin-related, various | 4 | 0.27-0.33 x |
| Chloroplast related, various | 5 | 0.28-0.48 x |
| Tetrapyrrole synthesis: Magnesium chelatase, uroporphyrinogen decarboxylase and glu-tRNA reductase | 4 | 0.31-0.44 x |
| Plastocyanin related | 2 | 0.2-0.49 x |
| Rubisco activase precursor | 1 | 0.16 x |
| Phytocyanin-related protein | 1 | 0.2 x |
| Thylakoid luminal 29.8 KDa protein and STOMAGEN | 2 | 0.39-0.41 x |
| Carbohydrate metabolism-related | 16 |  |
| **ARB-TMV TRANSGENIC PLANTS** | | |
| **Photosynthesis and carbohydrate metabolism related** | **278** |  |
| Chlorophyll a/b binding protein, various | 101 | 0.003-0.48 x |
| Photosystem I: 11,16 KDa, sub units IV A,V, N, VI, X, D-1, D-2, E-2, H-1,2,K,L,O,XI related proteins, | 56 | 0.06-0.49 x |
| Photosystem II:Lhcbm6, OEC, 22-23 kDa, sub units W, R, Q related | 26 | 0.21-0.5 x |
| Electron carriers: Plastocyanin and PGR5-1A related | 20 | 0.07-0.43 x |
| Chlorophyll synthesis related | 25 | 0.04-0.43 x |
| Miscellaneous | 9 | 0.17-0.47 x |
| Carbohydrate metabolism-related | 41 |  |
| **TMVi PLANTS** | | |
| **Photosynthesis and carbohydrate metabolism related** | **62** |  |
| LHC: Chlorophyll a-b binding protein and Lhcb4 | 9 | 0.19- 0.47 x |
| Ribulose bisphosphate carboxylase | 8 | 0.27- 0.43 x |
| ATP synthase protein | 3 | 0.33- 0.49 x |
| Photosystem I reaction center related | 4 | 0.3-0.49 x |
| PGR5-like protein 1A | 2 | 0.43- 0.47 x |
| Photosystem II reaction center related | 3 | 0.37- 0.47 x |
| Plastoquinone dehydrogenase | 3 | 0.39- 0.47 x |
| Carbonic anhydrase 1 | 4 | 0.21- 0.38 x |
| Alpha-glucan water dikinase | 4 | 0.27- 0.45 x |
| Ribose-5-phosphate isomerase | 3 | 0.43-0.45 x |
| Glycolysis: Phosphogluco mutase, G-6-P isomerase and pyruvate kinase | 3 | 0.43- 0.49 x |
| Pyruvate orthophosphate dikinase | 2 | 0.47- 0.5 x |
| Alcohol dehydrogenase 3 | 2 | 0.45- 0.49 x |
| Miscellaneous | 12 | 0.26- 0.49 x |
