# Supplementary material for: Multiple Different Defense Mechanisms Are Activated in the Young Transgenic Tobacco Plants Which Express the Full Length Genome of the Tobacco Mosaic Virus, and Are Resistant against this Virus
Source: PLoS One. 2014 Sep 22;9(9):e107778. doi: 10.1371/journal.pone.0107778 (PMC4171492; doi:10.1371/journal.pone.0107778)
Supplement: Table S11 — Photosynthesis and carbohydrate metabolism related up-regulated transcripts detected in the leaves of BRB-, ARB- transgenic and TMVi plants. (DOCX) [file pone.0107778.s014.docx]

| **Table S11. A list of up-regulated genes related to photosynthesis and carbohydrate metabolism in the BRB-, ARB-TMV transgenic and in TMVi plants.** | | |
| --- | --- | --- |
|  | **Total number of positive detections** | **Range of fold -change enhancement** |
| **BRB-TMV TRANSGENIC PLANTS** | | |
| **Photosynthesis and carbohydrate metabolism related** | **87** |  |
| Calvin cycle: aldolase and G-3-P dehydrogenase | 5 | 2-2.4 x |
| Chlrorespiration: PGR5-like A and alternative oxidase | 6 | 2- 4.6 x |
| PS I and PS II subunits: L,O,R, OEC protein and PsbP like proteins | 8 | 2- 3.1 x |
| NAD(P)H:plastoquinone dehydrogenase complex | 3 | 2.1-2.4 x |
| ATP synthase related | 2 | 2.7-3 x |
| Chloroplast related | 3 | 2- 2.3 x |
| (S)-2-hydroxy-acid oxidase, Peroxisomal | 1 | 3 x |
| Alpha-glucan water dikinase | 5 | 2.2- 8.2 x |
| Amylases: Iso-, alpha- and beta types | 9 | 2.0- 5.3 x |
| Sucrose phosphate synthase | 8 | 2.0- 3.4 x |
| Enzymes: carbohydrate metabolism related, various | 17 | 2.0 - 3.9 x |
| Miscellaneous | 20 | 2.0 – 4.8 x |
| **ARB-TMV TRANSGENIC PLANTS** | | |
| **Photosynthesis and carbohydrate metabolism related** | **52** |  |
| Starch degradation: 1,4-alpha-glucan-maltohydrolase, Iso amylase, beta-amylase and heteroglycan glucosidase 1 related | 5 | 2.2-3.8 x |
| Sucrose synthase 2, Fructose-1,6-bisphosphatase, Sucrose-6-phosphate synthase A and B related | 7 | 2.2-4.9 x |
| Inositol oxygenase 1 related | 1 | 4 x |
| Alcohol and aldehyde dehydrogenases related | 4 | 2.3-7.9 x |
| Branched chain alpha-keto acid and dihydrolipoamide  dehydrogenases related | 4 | 2.3- 6.2 x |
| Carbonic anhydrase related | 5 | 2.5-57.6 x |
| Enolase related | 3 | 2.1-2.6 x |
| UDP-glucuronate decarboxylase 3 | 1 | 2.5 x |
| Pyruvate and PEP carboxylase kinase | 2 | 2-3.7 x |
| Calvin cycle related, various | 5 | 2- 3.2 x |
| Photorespiration related, various | 4 | 2.1 x |
| Electron carriers: Ferredoxin, plastocyanin, cupredoxin and ATP synthase chain related | 6 | 2.1-2.9 x |
| SOUL heme-binding family protein | 3 | 2.1-2.6 x |
| Miscellaneous | 2 | 2.0-3.0 x |
| **TMVi PLANTS** | | |
| **Photosynthesis and carbohydrate metabolism related** | **5** |  |
| Beta-amylase | 2 | 2.7-2.9 x |
| Trehalose-6-phosphate phosphatase and L-lactate dehydrogenase | 2 | 2- 2.6 x |
| Chloroplast precursor protein | 1 | 2.7 x |
