# Supplementary material for: Multiple Different Defense Mechanisms Are Activated in the Young Transgenic Tobacco Plants Which Express the Full Length Genome of the Tobacco Mosaic Virus, and Are Resistant against this Virus
Source: PLoS One. 2014 Sep 22;9(9):e107778. doi: 10.1371/journal.pone.0107778 (PMC4171492; doi:10.1371/journal.pone.0107778)
Supplement: Table S12 — Cell division and DNA-binding related down-regulated transcripts detected in the leaves of BRB-, ARB- transgenic and TMVi plants. (DOCX) [file pone.0107778.s015.docx]

| **Table S12. A list of down-regulated genes related to cell division, cell organization, chromatin, DNA-repair and binding in the BRB-, ARB-TMV transgenic and in TMVi plants.** | | | | |
| --- | --- | --- | --- | --- |
|  | **Number of positive detections** | | | **Range of fold -enhancement** |
| **BRB-TMV TRANSGENIC PLANTS** | | | | |
| **Cell division and nucleus related** | | **30** |  | |
| Cyclin A,B and protein kinase like | | 16 | 0.18-0.42 x | |
| Peptidyl-prolyl cis-trans isomerase | | 4 | 0.31-0.40 x | |
| Mitotic spindle checkpoint protein | | 2 | 0.33-0.37 x | |
| Targeting protein for XKLP2 | | 3 | 0.35-0.49 x | |
| Nucleus: Nucleolar, GTPAse and transport factor | | 3 | 0.44-0.48 x | |
| Knolle gene | | 1 | 0.27 x | |
| Microtubule-binding protein TANGLED1 | | 1 | 0.47 x | |
| **ARB-TMV TRANSGENIC PLANTS** | | | | |
| **Cell division and organization related** | | **37** |  | |
| Cell division control protein 48 | | 2 | 0.43-0.47 x | |
| Peptidyl-prolyl cis-trans isomerase cyclophilin-type | | 1 | 0.15 x | |
| Cyclin-dependent protein kinase regulator | | 1 | 0.32 x | |
| Regulator: Plastid division Mind and chromosome condensation | | 2 | 0.45-0.47 x | |
| FtsZ-like protein precursor | | 1 | 0.46 x | |
| Tubulin: alpha, Beta type and microtubule related proteins | | 13 | 0.28-0.48 x | |
| Ankyrin repeat and annexin family protein | | 5 | 0.19-0.5 x | |
| Kinesin motor protein | | 3 | 0.41-0.48 x | |
| Harpin binding protein 1 | | 2 | 0.37-0.49 x | |
| Cyclin-like F-box | | 1 | 0.34 x | |
| Vesicle transport: Auxilin-like J domain | | 2 | 0.37-0.41 | |
| Miscellaneous | | 4 | 0.3-0.43 x | |
| **TMVi PLANTS** | | | | |
| **Cell division, Organisation and transport related** | | **5** |  | |
| Cyclin-dependent protein kinase | | 1 | 0.26 x | |
| Annexin related | | 4 | 0.4- 0.47 x | |
| **BRB-TMV TRANSGENIC PLANTS** | | | | |
| **Chromatin, DNA repair and binding related** | | **82** |  | |
| Histones: H1,H2A, H2B, H3 and H4 | | 54 | 0.24-0.48 x | |
| H/ACA ribonucleoprotein complex subunit 3-like protein | | 7 | 0.28-0.47 x | |
| High mobility group B protein 6 | | 4 | 0.36-0.49 x | |
| Nucleosome assembly protein 1 - like protein 2 | | 3 | 0.31-0.43 x | |
| Topoisomerase II | | 4 | 0.22-0.44 x | |
| Delta DNA polymerase 4 and DNA gyrase | | 2 | 0.37-0.47 x | |
| DNA repair: DNA mismatch repair and Photolyase related | | 4 | 0.42-0.47 x | |
| DNA binding: vp1, translin, deacetylase, and Origin recognition complex | | 4 | 0.21-0.48 x | |
| **ARB-TMV TRANSGENIC PLANTS** | | | | |
| **Chromatin, DNA repair and binding related** | | **13** |  | |
| DNA photolyase | | 2 | 0.37-0.40 x | |
| UvrB/uvrC motif family protein | | 3 | 0.27-0.42 x | |
| DNA binding, various | | 5 | 0.08-0.44 x | |
| GCN5-related N-acetyltransferase | | 1 | 0.38 x | |
| H2B histone-fold-like protein | | 1 | 0.36 x | |
| Cyclase family protein | | 1 | 0.45 x | |
| **TMVi PLANTS** | | | | |
| **Chromatin, DNA repair and binding related** | | **4** |  | |
| DNA binding protein | | 2 | 0.45- 0.48 x | |
| Transposase | | 1 | 0.5 x | |
| Nuclease | | 1 | 0.33 x | |
