# Supplementary material for: Multiple Different Defense Mechanisms Are Activated in the Young Transgenic Tobacco Plants Which Express the Full Length Genome of the Tobacco Mosaic Virus, and Are Resistant against this Virus
Source: PLoS One. 2014 Sep 22;9(9):e107778. doi: 10.1371/journal.pone.0107778 (PMC4171492; doi:10.1371/journal.pone.0107778)
Supplement: Table S13 — Cell division and DNA-binding related up-regulated transcripts detected in the leaves of BRB-, ARB- transgenic and TMVi plants. (DOCX) [file pone.0107778.s016.docx]

| **Table S13. A list of up-regulated genes related to cell division, cell organization, chromatin, DNA binding and repair proteins related in the BRB-, ARB-TMV transgenic and in TMVi plants.** | | |
| --- | --- | --- |
|  | **Total number of positive detections** | **Range of fold -change enhancement** |
| **BRB-TMV TRANSGENIC PLANTS** | | |
| **Cell division, organization and transport related** | **18** |  |
| cell Organisation: Annexin, HIPL2, 2-A15 and tubulin | 11 | 2.1-6.5 x |
| Ankyrin repeat family protein | 2 | 3.2-3.4 x |
| cell division: yippee-like, RCC1 and 27 homolog B proteins | 3 | 2-6.2 x |
| Syntaxin 125 | 1 | 2.7 x |
| Myosin-13, putative | 1 | 3.1 x |
| **ARB-TMV TRANSGENIC PLANTS** | | |
| **Cell division, organization and transport related** | **17** |  |
| Cyclin related | 3 | 2.1-2.4 x |
| Cell cycle check points: MAD2 and RAD9A like | 2 | 2-149 x |
| Cell division: MFP1, Glucose inhibited protein and Phytosulfokine peptide related | 3 | 2-2.4 x |
| Motor proteins; Kinesin and myosin | 3 | 2-2.1 x |
| Actin binding related | 2 | 2.1-2.4 x |
| Annexin related | 2 | 2.4-2.9 x |
| Tubulin related | 1 | 3.7 x |
| Powdery mildew resistance protein 5 | 1 | 3.3 x |
| **TMVi PLANTS** | | |
| **Cell division, organization and transport related** | **5** |  |
| Cell cycle checkpoint control protein | 1 | 138 x |
| Miscellaneous | 4 | 2-2.9 x |
| **BRB-TMV TRANSGENIC PLANTS** | | |
| **Chromatin and DNA binding and repair proteins related** | **11** |  |
| UvrB/uvrC motif family protein | 2 | 2.9 x |
| GCN5-related N-acetyltransferase (GNAT) | 3 | 2.4-4.2 x |
| RXT3-like protein, putative | 1 | 2.2 x |
| 3' exoribonuclease | 2 | 2.3-2.4 x |
| Histone H2B | 1 | 3 x |
| Type II inositol-1,4,5-trisphosphate 5-phosphatase 12 | 1 | 2 x |
| DNA methyltransferase 1-associated protein | 1 | 2.2 x |
| **ARB-TMV TRANSGENIC PLANTS** | | |
| **Chromatin and DNA binding and repair proteins related** | **16** |  |
| DNA binding related, various | 6 | 2.1-2.8 x |
| Histone protein related | 4 | 2-2.8 x |
| ULTRAPETALA1 | 3 | 2.2-2.7 x |
| GCN5-related N-acetyltransferases related | 2 | 2.5-2.6 x |
| NADH dehydrogenase 1 | 1 | 2.3 x |
| **TMVi PLANTS** | | |
| **Chromatin and DNA binding and repair proteins related** | **1** |  |
| NAP1-related protein | 1 | 2 x |
