# Supplementary material for: Multiple Different Defense Mechanisms Are Activated in the Young Transgenic Tobacco Plants Which Express the Full Length Genome of the Tobacco Mosaic Virus, and Are Resistant against this Virus
Source: PLoS One. 2014 Sep 22;9(9):e107778. doi: 10.1371/journal.pone.0107778 (PMC4171492; doi:10.1371/journal.pone.0107778)
Supplement: Table S14 — Different up-regulated transcripts in the BRB-TMV transgenic plants after subtracting the up-regulated transcripts from other ARB-TMV, TMVi and different VRS expressing transgenic tobacco plants (HcPro, AC2 and P25). (DOCX) [file pone.0107778.s017.docx]

| **Table S14**. **A list of the up-regulated genes related to different functional groups in the BRB-TMV transgenic plants after subtracting the up-regulated genes of the ARB-TMV transgenic and TMVi plants, and other VSRs expressing (HcPro, Ac2 and P25) transgenic plants.** | | |
| --- | --- | --- |
|  | **Total number of positive detections** | **Range of fold -change enhancement** |
| **BIOTIC AND ABIOTIC STRESS RELATED** | **64** |  |
| Heat shock proteins: 175, 26.5 KDa, chaperone and DnaJ 20 related | 8 | 2.2-3 x |
| Cold acclimation protein WCOR413-like | 3 | 2.6-2.8 x |
| Dehydrin like protein | 7 | 2.2-3.2 x |
| Elicitor responsible protein | 3 | 2.9-3.5 x |
| Endochitinase B precursor | 2 | 2-4 x |
| Glycine rich protein | 6 | 3-4.6 x |
| HR related: BAX type inhibitors and Band 7 family | 5 | 2x |
| Osmotin 34 precursor | 7 | 3- 14.2 x |
| Pollen coat-like protein | 2 | 2.9-3.1 x |
| VAMP protein SEC22 | 2 | 2.5 x |
| Wound-induced protein 1 | 5 | 2.2- 10 x |
| Abscisic acid stress Ripening related | 3 | 2 x |
| Xenobiotics: 3-HBA dehydrogenase and Carboxyesterase 18 | 2 | 2 x |
| Defense related: PR proteins, Thaumatin and tetracyclin related | 4 | 2-3.8 x |
| Miscellaneous | 5 | 2.1-5.1 x |
| **ROS related** | **34** |  |
| Catalase (CAT-1) | 5 | 2.5-2.9 x |
| Cytochrome 450 related | 6 | 2-4.3 x |
| Glutathione peroxidase | 2 | 2.1-2.4 x |
| Glutathione S-transferase | 9 | 2-2.3 x |
| Peroxidase related | 8 | 2.1-5.8 x |
| Thioredoxin-like protein | 2 | 5.2-5.7 x |
| Oxidoreductase family protein | 1 | 2.4 x |
| Membrane steroid-binding protein 1 | 1 | 2.2 x |
| **Protein synthesis, degradation and amino acid metabolism related** | **87** |  |
| Ribosomal proteins: 30S, 60S and S10 | 3 | 2-3.8 x |
| Autophagy 8c protein | 4 | 2.1-3.4 x |
| Ubiquitin: Ligases, conjugating and carrier like proteins | 13 | 2-2.8 x |
| Translation initiation factor SUI1 | 4 | 2-3.1 x |
| Proteases: Subtilases, Xaa-Pro aminopeptidase 2,  Peptidase- M48, S1, S6 and FTsH related | 10 | 2.1-6.3 x |
| C3HC4-type RING finger family protein | 4 | 2-2.2 x |
| F-box family related | 6 | 2-4.1 x |
| Cathepsin B-like cysteine protease and Aspartyl protease | 2 | 2-2.1 |
| Aminopeptidase | 1 | 2.5 x |
| Polyubiquitin protein | 2 | 2-2.2 x |
| Pyrrolidone-carboxylate peptidase | 3 | 2.4 x |
| Serine carboxypeptidase-like 40 | 2 | 2.2-2.4 x |
| SKP1-interacting partner 1 | 1 | 2.1 x |
| Serine/threonine protein kinase SAPK8-like protein | 4 | 2-5.4 x |
| Post translational modifications: kinases, OBP3-responsive, CBL-interacting protein kinase, CIPK 1 and PPR repeat-containing proteins | 14 | 2-6.7 x |
| Proteinase inhibitors | 3 | 2-6.1 x |
| Amino acid synthesis: Cysteine synthase, Glutamate decarboxylase and Immunophilin | 6 | 2-3.6 x |
| Protein targeting, various | 3 | 2.2-2.6 x |
| Branched-chain amino acid aminotransferase | 1 | 2.3 x |
| Beta-N-acetylhexosaminidase | 1 | 2.3 x |
| **Nucleotide metabolism related** | **6** |  |
| Adenylate kinase family protein | 2 | 2 x |
| Uricase | 3 | 2.1-2.2 x |
| Inositol-1,4,5-triphosphate-5-phosphatase (5PT4) | 1 | 2.1 x |
| **Photosynthesis and carbohydrate metabolism related** | **53** |  |
| PGR5-like A and Alternative oxidase | 5 | 2.1-2.4 x |
| Photosystem I subunit L | 2 | 2-2.2 x |
| Photosystem II subunit R, OEC and PPL2 | 5 | 2- 3.11 x |
| NAD(P)H:plastoquinone dehydrogenase complex | 3 | 2.1-2.3 x |
| Calvin cycle: Aldalose and GAP | 2 | 2.3-2.4 x |
| (S)-2-hydroxy-acid oxidase, peroxisomal | 1 | 3.2 x |
| Chloroplast related | 3 | 2-2.3 x |
| ATP synthase protein I -related | 1 | 2.7 x |
| Starch degradation: Alpha-glucan water dikinase, Beta-amylase, Tyrosine phosphatase-like and Alpha-glucan phosphorylase | 8 | 2.2-3.8 x |
| Starch branching enzyme 2.1 and 2.2 | 2 | 2-2.4 x |
| DIN10 (Dark inducible 10) hydrolase | 4 | 2-4.7 x |
| Sucrose synthesis: Sucrose-phosphate synthase, Fructokinase-like and Fructose-1,6-bisphosphatase | 6 | 2.1-3.9 x |
| Sucrose degradation: Sucrose synthase 2, 3 | 2 | 2 x |
| Malate dehydrogenase | 2 | 2 x |
| NADP-dependent malic enzyme | 2 | 3.9 x |
| Xylose isomerase | 1 | 2 x |
| 3-chloroallyl aldehyde dehydrogenase/ aldehyde dehydrogenase (NAD) | 1 | 2.1 x |
| Glycolysis: Phosphogluco mutase and G-6-P isomerase | 2 | 2-4.4 x |
| Glucosamine/galactosamine-6-phosphate isomerase family protein | 1 | 2.2 x |
| **Transcription factor related** | **46** |  |
| Bell-like homeodomain protein 2 | 7 | 2.1-3.8 x |
| DC1 domain-containing protein | 4 | 4.2-4.7 x |
| EIN3 (Ethylene-Insensitive 3) | 2 | 2.4-2.5 x |
| Homeobox-leucine zipper protein, various | 10 | 2.2-4 x |
| Zinc finger: AN1 like, B-box, ISAP1, DNL and CCHH family proteins | 12 | 2-3.7 x |
| Transcription factors, various | 11 | 2-4.4 x |
| **RNA binding and processing related** | **6** |  |
| RNA processing: U6 sn RNA and ERI1 exoribonuclease | 3 | 2-3.8 x |
| RNA binding , various | 3 | 2-3.4 x |
| **Secondary metabolism related** | **11** |  |
| 5-epi-aristolochene synthase | 2 | 2-6.2 x |
| Iron-sulfur assembly protein IscA | 2 | 2.1-2.3 x |
| One carbon metabolism, various | 3 | 2-2.2 x |
| FMO-like monooxygenase | 1 | 2.3 x |
| NADP-dependent oxidoreductase, putative | 1 | 2.4 x |
| Scenescence related gene 1 | 1 | 3 x |
| Dihydroflavonol 4-reductase | 1 | 2.9 x |
| **Signaling related** | **14** |  |
| Calmodulin-related protein | 4 | 2-2.2 x |
| GTP-binding family protein | 2 | 2.3-3.3 x |
| Mitogen associate protein kinase 3 | 5 | 2.3-3.8 x |
| CLAVATA1 precursor | 1 | 2.2 x |
| Leucine rich repeat family protein | 1 | 2.2 x |
| Patched protein homolog 1 | 1 | 2.1 x |
| **Transporters related** | **27** |  |
| Ca2+/H+ exchanger | 3 | 4.2-4.7 x |
| Metal transporter | 5 | 2-3.2 x |
| Ammonium transmembrane transporter | 2 | 4.7- 7.9 x |
| Cyclic nucleotide-gated ion channel 2 | 2 | 2.4-2.5 x |
| Nucleoside transporter, putative | 2 | 2.2-2.5 x |
| ABC family | 2 | 2.3-3.8 x |
| Sugar transporter | 2 | 2.7-3.6 x |
| Various transporters | 9 | 2-3.1 x |
| **Cell wall and membrane related** | **26** |  |
| Beta galactosidase and Beta-1,3-glucanase | 4 | 2.1-12.2 x |
| Pectin methylesterase | 4 | 2-2.4 x |
| Cell wall protein | 2 | 2.9 4.1 x |
| Expansin-like B1 | 2 | 2.1-2.3 x |
| Cell wall degradation related: BURP, LEXY2 and Xylan 1,4-beta-xylosidase | 3 | 2.2-3.5 x |
| Cellulose synthase | 1 | 2.3 x |
| NAD dependent epimerase | 1 | 2.1 x |
| Xyloglucan endotransglucosylase-hydrolase XTH7 | 1 | 2.3 x |
| Remorin 2 (rem-2) protein | 4 | 2.3-2.6 x |
| Miscellaneous | 4 | 2.2-2.5 x |
| **Hormones and Development related** | **39** |  |
| Auxin associated family protein | 2 | 11.5-15.1 x |
| BYPASS 1 protein | 2 | 2 x |
| Senescence-associated protein | 7 | 2.2-9 x |
| Nodulin MtN3 family protein | 2 | 2.4-2.6 x |
| Dormancy associated protein | 2 | 11-12.7 x |
| GIGANTEA protein | 1 | 3 x |
| Ethylene synthesis:1-aminocyclopropane-1-carboxylate oxidase,2OG-Fe(II) oxygenase and Induced related | 10 | 2.1-3.3 x |
| Auxin: Dormancy and Induced proteins | 5 | 2-10.7 x |
| Abscisic acid: synthesis, degradation and Induced related | 5 | 2-4 x |
| Gibberellin-responsive protein | 1 | 5.1 x |
| Miscellaneous | 2 | 2.4-2.5 x |
| **Chromatin and DNA binding related** | **9** |  |
| UvrB/uvrC motif family protein | 2 | 2.9 x |
| Exonuclease related | 2 | 2.4-2.47 x |
| Histone: Histone 2b,acetylation and de acetylation | 3 | 2.2-4.2 x |
| Type II inositol-1,4,5-trisphosphate 5-phosphatase 12 | 1 | 2 x |
| DNA methyltransferase 1-associated protein | 1 | 2.2 x |
| **Lipid metabolism related** | **27** |  |
| GDSL-motif lipase/hydrolase family protein | 4 | 2-2.8 x |
| Lipid transfer protein and associated proteins | 6 | 2.1-4.4 x |
| Phospholipase related | 5 | 2.3-3.6 x |
| Diacylglycerol acyltransferase | 2 | 6 x |
| Saposin B domain-containing protein | 2 | 2.5-2.7 x |
| stAR-related lipid transfer protein 7, predicted | 2 | 2.1-2.6 x |
| Miscellaneous | 6 | 2-7.1 x |
| **Metal binding proteins related** | **27** |  |
| Aluminum-induced protein | 3 | 2-2.6 x |
| Metallothionein-like protein | 19 | 2.2-3 x |
| Metallo-beta-lactamase family protein | 2 | 2.1-2.2 x |
| Metal ion binding protein | 1 | 2.3 x |
| Stem-specific protein TSJT1 | 2 | 2.1-2.2 x |
| **Kinases and phosphatases related** | **13** |  |
| Acid phosphatase 1 precursor | 2 | 2.2-2.6 x |
| Phosphatases, various | 4 | 2-2.4 x |
| Kinases, various | 7 | 2-7.2 x |
| **Glucosyle transferses related** | **4** |  |
| Glucosyl transferases | 4 | 2.2-2.7 x |
| **Electron transport related** | **6** |  |
| Integral membrane protein, putative | 2 | 2-2.1 x |
| NADH dehydrogenase subunit 5, putative | 3 | 2-3.7 x |
| ATP synthase | 1 | 2.1 x |
| **Cell division and Organisation related** | **7** |  |
| Annexin | 3 | 2.8-5.1 x |
| Beta-5 tubulin | 1 | 3.2 x |
| Cell division cycle protein 27 homolog B and Regulator of chromosome condensation (RCC1) family protein | 2 | 2-2.3 x |
| Phloem protein | 1 | 2.2 x |
| **Functions not assigned** | **24** |  |
| Interesting and miscellaneous | 24 | 2-18 x |
| **Unknowns** | **165** |  |
| Miscellaneous | 165 | 2-38 x |
