# Supplementary material for: Multiple Different Defense Mechanisms Are Activated in the Young Transgenic Tobacco Plants Which Express the Full Length Genome of the Tobacco Mosaic Virus, and Are Resistant against this Virus
Source: PLoS One. 2014 Sep 22;9(9):e107778. doi: 10.1371/journal.pone.0107778 (PMC4171492; doi:10.1371/journal.pone.0107778)
Supplement: Table S15 — Different down-regulated transcripts in the BRB-TMV transgenic plants after subtracting the up-regulated transcripts from other ARB-TMV, TMVi and different VRS expressing transgenic tobacco plants (HcPro, AC2 and P25). (DOCX) [file pone.0107778.s018.docx]

| **Table S15. A list of the down-regulated genes related to different functional groups in the BRB-TMV transgenic plants after subtracting the down-regulated genes of ARB-TMV transgenic and TMVi plants and of other VSRs expressing (HcPro, AC2 and P25) transgenic plants.** | | |
| --- | --- | --- |
|  | **Total number of negative detections** | **Range of fold -change enhancement** |
| **BIOTIC AND ABIOTIC STRESS RELATED** | **48** |  |
| Heat shock proteins: DnaJ family,17.8 kDa and chaperons | 13 | 0.24- 0.49 x |
| Arabinogalactan-protein precursor | 4 | 0.41-0.46 x |
| ATP binding, various | 5 | 0.29-0.48 x |
| Endochitinase PR4 precursor | 2 | 0.42-0.47 x |
| Defense proteins: Major latex protein, Thaumatin, Thioinin and Osmotin precursor related | 6 | 0.27- 0.49 x |
| Phosphosulfolactate synthase-related protein | 2 | 0.39-0.47 x |
| Stress related, various | 16 | 0.19-0.49 x |
| **ROS and oxidoreductase related** | **15** |  |
| Cytochrome p450 related | 7 | 0.18-0.48 x |
| Ascorbate peroxidase 1 | 2 | 0.43-0.44 x |
| Peroxisomal membrane 22 kDa family protein | 2 | 0.25 x |
| Oxidase/Oxidoreducatse | 4 | 0.23-0.47 x |
| **Protein synthesis, degradation and amino acid related** | **676** |  |
| Ribosomal proteins: 16 S and 18 S | 4 | 0.29-0.39 x |
| 10 and 20 KDa chaperon | 2 | 0.29 x |
| 28 kDa small subunit ribosomal protein | 4 | 0.3-0.5 x |
| 3 MAT3 (methionine adenosyl transferase 3) | 2 | 0.39-0.48 x |
| 3-phosphoshikimate 1-carboxyvinyltransferase | 3 | 0.46-0.49 x |
| 40S ribosomal protein, various | 189 | 0.22-0.5 x |
| 60S acidic ribosomal protein, various | 356 | 0.21-0.49 x |
| Acidic ribosomal protein P1a-like | 8 | 0.27-0.48 x |
| Ubiquitin related, various | 14 | 0.27-0.49 x |
| Protein synthesis: Initiation and elongation related | 39 | 0.28-0.49 x |
| Chaperons | 13 | 0.28-0.46 x |
| ketol-acid reductoisomerase | 3 | 0.40-0.48 x |
| Methionine synthase | 4 | 0.42-0.46 x |
| Phospho-2-dehydro-3-deoxyheptonate aldolase 2 | 3 | 0.27-0.44 x |
| Protein degradation: Subtilases, Aminopeptidase, AAA type, and cysteine protease | 8 | 0.38-0.49 x |
| Acetolactate synthase 2 | 3 | 0.41-0.48 x |
| Protein targeting related | 7 | 0.32-0.49 x |
| Amino acid metabolism, various | 10 | 0.24-0.49 x |
| Post translational modification | 4 | 0.21-0.47 x |
| **Photosynthesis and carbohydrate metabolism related** | **18** |  |
| Chloroplast related, various | 5 | 0.28-0.48 x |
| Ferridoxin related | 3 | 0.27-0.33 x |
| NADP-dependent glyceraldehyde-3-phosphate dehydrogenase | 3 | 0.2-0.22 x |
| Plastocyanin related | 2 | 0.2-0.49 x |
| Rubisco carboxylase | 2 | 0.16-0.36 x |
| Miscellaneous | 3 | 0.2-0.39 x |
| **RNA processing and Binding related** | **31** |  |
| Exoribonucleases | 2 | 0.43-0.44 x |
| RNA binding: 31, 33KDa ribonucleo protein, Glycine rich proteins | 6 | 0.42-0.49 x |
| Poly(A)-binding protein | 4 | 0.2-0.35 x |
| DEAD box RNA helicase | 4 | 0.34-0.48 x |
| Small nuclear ribonucleoprotein | 7 | 0.4-0.48 x |
| ATP binding protein | 3 | 0.38-0.44 x |
| Fibrillarin | 2 | 0.33-0.39 x |
| Miscellaneous | 3 | 0.44-0.5 x |
| **Transcription factors related** | **23** |  |
| C2H2-like zinc finger protein | 3 | 0.41-0.45 x |
| DNA binding, various | 4 | 0.23-0.44 x |
| MAR-binding protein | 2 | 0.3-0.37 x |
| Miscellaneous: 98b, B3, BHLH,E2F,TUB, NOP-56,ARR9 and Yabby family transcription factors | 9 | 0.23-0.45 x |
| Transcription regualtors, various | 5 | 0.21-0.49 x |
| **Assimilation related** | **5** |  |
| Adenylyl-sulfate reductase | 4 | 0.23-0.38 x |
| 3-phosphoadenosine 5-phosphosulfate synthetase 2 | 1 | 0.36 x |
| **Signalling related** | **19** |  |
| G protein beta subunit-like protein | 15 | 0.23-0.47 x |
| Leucin rich repeat protein | 1 | 0.49 x |
| Mapk kinase | 1 | 0.47 x |
| Miscellaneous | 2 | 0.35 x |
| **Transporters related** | **18** |  |
| Metabolite transporters: TOM 6 & 7 | 3 | 0.38-0.47 x |
| Sugar: 34,36 kDa porins and Voltage-dependent anion channel | 4 | 0.46-0.49 x |
| ABC type | 2 | 0.4-0.43 x |
| Patellin-4 | 2 | 0.32 x |
| Secretory carrier membrane protein (SCAMP) | 2 | 0.47-0.48 x |
| Miscellaneous | 5 | 0.31-0.48 x |
| **Nucleotide metabolism related** | **15** |  |
| AICARFT/IMPCHase bienzyme family protein | 2 | 0.42 x |
| Deoxynucleoside kinase family | 2 | 0.45 x |
| Nucleoside diphosphate kinase 1 | 5 | 0.36-0.44 x |
| Nucleolar protein 6-like, predicted | 2 | 0.45-0.48 x |
| Adenine phosphoribosyltransferase 5 | 1 | 0.28 x |
| Miscellaneous | 3 | 0.29-0.45 x |
| **Cell division and organisation related** | **44** |  |
| Ankyrin repeat family protein | 5 | 0.39-0.48 x |
| Cyclin- A and B type kinase | 10 | 0.18-0.38 x |
| Actin related: ADF7 and ABI | 2 | 0.31-0.47 x |
| Tubulin: alpha and beta | 3 | 0.34-0.42 x |
| Kinesin like | 8 | 0.26-0.40 x |
| Microtubule-associated protein | 3 | 0.27-0.47 x |
| Mitotic spindle checkpoint protein | 2 | 0.33-0.37 x |
| Peptidyl-prolyl cis-trans isomerase | 4 | 0.31-0.40 x |
| Syntaxin related | 2 | 0.28-0.48 x |
| Targeting protein for XKLP2 | 3 | 0.39-0.45 x |
| Myosin-9 | 1 | 0.46 x |
| Knolle (kn gene) | 1 | 0.27 x |
| **Chromatin, DNA binding and repair related** | **63** |  |
| DNA topoisomerase II | 3 | 0.3-0.44 x |
| H/ACA ribonucleoprotein complex subunit 3-like protein | 6 | 0.28-0.47 x |
| Histones: 1A, 2A, 2B, VP1, translin and H4 related | 39 | 0.32-0.48 x |
| Nucleosome assembly protein 1 - like protein 2 | 3 | 0.31-0.43 x |
| SWIB complex BAF60b domain-containing protein | 2 | 0.44-0.45 x |
| DNA repair: mismatch repair and photolyase | 3 | 0.42-0.47 x |
| DNA gyrase subunit A and polymerase | 2 | 0.37-0.47 x |
| Chromomethylase 3 (CMT3) | 2 | 0.39-0.47 x |
| High mobility group B protein 6 | 2 | 0.48-0.49 x |
| Deacetylase-like protein | 1 | 0.21 x |
| **Lipid metabolism related** | **24** |  |
| 14 kDa proline-rich protein DC2.15 precursor | 3 | 0.07-0.48 x |
| Beta-hydroxyacyl-ACP dehydratase | 5 | 0.43-0.48 x |
| Biotin carboxyl carrier protein of acetyl-CoA carboxylase | 4 | 0.42-0.46 x |
| GDSL-motif lipase/hydrolase | 2 | 0.41-0.45 x |
| Lipid binding protein, putative | 3 | 0.23-0.36 x |
| Miscellaneous | 7 | 0.25-0.47 x |
| **Carbohydrate metabolism related** | **14** |  |
| Phosphoenolpyruvate carboxylase | 4 | 0.41-0.47 x |
| Pyruvate kinase | 3 | 0.39-0.41 x |
| NAD-malate dehydrogenase and malic enzyme related | 4 | 0.33-0.5 x |
| Inositol monophosphatase 3, fructose-6-phosphate 1-phosphotransferase and phosphopyruvate related | 3 | 0.45-0.49 x |
| **Hormones and methylation related** | **3** |  |
| Auxin-responsive family protein | 1 | 0.32 x |
| Snakin-1 (SN1) | 1 | 0.48 x |
| SAM synthatase | 1 | 0.4 x |
| **Kinases, Glucosyl transferases and metal binding related** | **5** |  |
| Nodulation receptor kinase | 2 | 0.27-0.41 x |
| Ferritin-1 protein | 2 | 0.41-0.48 x |
| Metal transport system membrane protein | 1 | 0.47 x |
| Galactomannan galactosyltransferase | 1 | 0.42 x |
| UDP-rhamnose | 1 | 0.21 x |
| UDP-glucoronosyl/UDP-glucosyl transferase | 1 | 0.42 x |
| **Cell wall and development related** | **28** |  |
| Extensin protein | 5 | 0.35-0.42 x |
| Pectate lyase family protein | 1 | 0.47 x |
| Pectate methyl esterase | 1 | 0.37 x |
| Beta glucanase: Beta-1,3-glucanase and Endo-beta-1,4-glucanase | 2 | 0.45-0.48 x |
| Glycosyl hydrolase | 2 | 0.21-0.43 x |
| Miscellaneous | 2 | 0.32-0.44 x |
| WD-40 repeat protein | 4 | 0.24-0.5 x |
| Dem protein-related | 2 | 0.41-0.45 x |
| DAG related | 1 | 0.47 x |
| Pale cress (PAC) protein | 2 | 0.29-0.39 x |
| Endosperm and embryo defective ( EDE1 and emb1923 ) | 2 | 0.34-0.48 x |
| Miscellaneous | 4 | 0.39-0.46 x |
| **Functions not assigned** | **20** |  |
| Miscellaneous | 20 | 0.11-0.49 x |
| **Unknowns** | **162** |  |
| Unknowns | 162 | 0.21-0.49 x |
