# Supplementary material for: Multiple Different Defense Mechanisms Are Activated in the Young Transgenic Tobacco Plants Which Express the Full Length Genome of the Tobacco Mosaic Virus, and Are Resistant against this Virus
Source: PLoS One. 2014 Sep 22;9(9):e107778. doi: 10.1371/journal.pone.0107778 (PMC4171492; doi:10.1371/journal.pone.0107778)
Supplement: Table S16 — Primers used in RT-qPCR experiment for validation of microarray data. (DOCX) [file pone.0107778.s019.docx]

**Table S16. Overview of RT-qPCR conditions according to the BIOMED central MIQE precise file format**

| **Sample/Template** | **details** |
| --- | --- |
| Source | Wild type, whole TMVexpressing transgenic and TMV infected wild type tobacco plants |
| Method of preservation | Liquid N2 and storage at -80 C |
| Storage time (if appropriate) | Stored frozen less than six months |
| Handling | frozen |
| Extraction method | Trisure (Bioline,UK) |
| RNA: DNA-free | DNAse I treatment (Promega RQ1 RNase free-DNAseI) |
| Concentration | Nanodrop |
| RNA: integrity | Agilent\2100 bioanalyzer |
| Inhibition-free | N/A |
| **Assay optimisation/validation** |  |
| Accession number | N/A |
| Amplicon details | See below |
| Primer sequence | see below |
| *Probe sequence** | No probes |
| *In silico* | Primer3 program used for designing primers |
| empirical | 0.2uM primer concentration/55 -60 C annealing temperature |
| Priming conditions | oligo-dT used for cDNA synthesis |
| PCR efficiency | Tested in conventional PCR |
| Linear dynamic range | Variable for different amplicons |
| Limits of detection | N/A |
| Intra-assay variation | N/A |
| **RT/PCR** |  |
| Protocols | See methods section, 3ul of cDNA with 3.3ng/ul concentration, 25 ul total volume |
| Reagents | Fermentas, EPO441-Reverse transciptase; Fermentas,K0242- Maxima SYBR Green/Fluorescein RT-qPCR Master Mix (2X) |
| Duplicate RT | 3 biological replicates S.E shown in table 1 |
| NTC | Melt curves checked and clean |
| NAC | Variable for different amplicons |
| Positive control | Same reference gene used in different runs |
| Data analysis |  |
| Specialist software | Bio-RAD iQ5 |
| Statistical justification | 3 biological replicates |
| Transparent, validated normalisation | Normalized with the reference gene |

**Primers used in the qRT-PCR for validating the microarray expression profile**

**Primers for differentially expressed genes**

**Primers for differentially expressed genes**

**BRB-TMV**

EB683763: P-rich protein NtEIG-C29

RIGHT PRIMER 5’-CGTCCCCTAGAAAACCCTGT -3’

LEFT PRIMER 5’-CATCTGGCTTCCAGTGTCCT-3’

BP128776: DNAJ heat shock protein

RIGHT PRIMER 5’- CTACCGGTGTTTTGGGAGAA-3’

LEFT PRIMER 5’- TGCAAAAAGAAGCACGAATG -3’

CV018266: 60s acidic ribosomal protein-like protein

RIGHT PRIMER 5’- CCCATGTCATCATCAGATTCC -3’

LEFT PRIMER 5’- GCTGCTTAGGCAAGACAACC -3’

DV158570: 40S ribosomal protein S8

RIGHT PRIMER 5’- GCAACAGTTAAGCAGCACCA -3’

LEFT PRIMER 5’- AGAAGAAGGGGCCTGCTAAG -3’

EB683199: 60S ribosomal protein L35

RIGHT PRIMER 5’- CTTTCGGATGGGGAAGTACA -3’

LEFT PRIMER 5’- GCAATGGCAAGAATCAAGGT -3’

**ARB-TMV**

EB438730: Dicer-2 like

RIGHT PRIMER 5’-ACCTGAGATTCCACGATGCT-3’

LEFT PRIMER 5’-ACACCGTCAGCTTCAACCTC-3’

EH620111: Pathogenesis-related protein 1B precursor

RIGHT PRIMER 5’-CAT GCC CAA AAC TCT CAA CA-3’

LEFT PRIMER 5’-CCT AGC ACA TCC AAC ACG AA-3’

EH617029: WRKY transcription factor-30

RIGHT PRIMER 5’-TGTGGATGTGATCACCAGAA-3’

LEFT PRIMER 5’-TGAATTTCACTCGCAGCTTG-3’

CV017513: Chlorophyll a-b binding protein 3A

RIGHT PRIMER 5’-CAGGCGTTGTTGTTAACTGG -3’

LEFT PRIMER 5’- GTCGACCACTCTACCTGGTG-3’

EH620344: F box related protein

RIGHT PRIMER 5’-AGAATATATAAATACAACGGCAAAAAG -3’

LEFT PRIMER 5’-TCTTGCTGTTTGTAGTCCCTTG -3’

**TMVi**

EH620111: Pathogenesis-related protein 1B precursor

RIGHT PRIMER 5’-CAT GCC CAA AAC TCT CAA CA-3’

LEFT PRIMER 5’-CCT AGC ACA TCC AAC ACG AA-3’

EB643469: 60s Acidic ribosomal protein

RIGHT PRIMER 5’-CGTCACCGGTATTAGGAGGA -3’

LEFT PRIMER 5’-TGCTTGTTTGATCCTTCACG -3’

EH620344: F box related protein

RIGHT PRIMER 5’-AGAATATATAAATACAACGGCAAAAAG -3’

LEFT PRIMER 5’-TCTTGCTGTTTGTAGTCCCTTG -3’

CV017513: Chlorophyll a-b binding protein 3A

RIGHT PRIMER 5’-CAGGCGTTGTTGTTAACTGG -3’

LEFT PRIMER 5’- GTCGACCACTCTACCTGGTG-3’

TA12913_4097: Pollen coat like protein

RIGHT PRIMER 5’- AAACGCAGAAGTTCTCACCAA -3’

LEFT PRIMER 5’- CCGGAGACATCCATAAGCAA -3’

**Primers for reference genes**

**BRB-TMV**

EB450395: ARPC3 (actin-related protein C3) protein like

FORWARD PRIMER 5’-CGCCGGTGAAATTACCTCT -3’

REVERSE PRIMER 5’-AAG TTCTGCAGCCTTCAAGC -3’

**ARB-TMV**

EH623128: 4,5-DOPA dioxygenase extradiol-like protein

RIGHT PRIMER 5’- GGCAGATCCAGACCCAATTA -3’

LEFT PRIMER 5’- GGGTGAAGGAAGTTCTCACG -3’

**TMVi**

BP534665: Citrate synthase

RIGHT PRIMER 5’- AACACCCCTAAATTGCGACA -3’

LEFT PRIMER 5’- GAGCTCTTGGCATTTGCTCT -3’
